# Biospeleothems Formed by Fungal Activity During the Early Holocene in the “Salar de Uyuni”

**DOI:** 10.3389/fmicb.2022.913452

**Published:** 2022-06-23

**Authors:** Angélica Anglés, Qitao He, Laura Sánchez García, Daniel Carrizo, Nuria Rodriguez, Ting Huang, Yan Shen, Ricardo Amils, David C. Fernández-Remolar

**Affiliations:** ^1^State Key Laboratory of Lunar and Planetary Sciences, Macau University of Science and Technology, Macau, China; ^2^China National Space Administration (CNSA) Macau Center for Space Exploration and Science, Macau, China; ^3^Blue Marble Space Institute of Science, Seattle, WA, United States; ^4^Centro de Astrobiología Instituto Nacional de Tecnica Aeroespacial - Consejo Superior de Investigaciones Científicas (INTA-CSIC), Madrid, Spain; ^5^Carl Sagan Center, The SETI Institute, Mountain View, CA, United States

**Keywords:** karst, fungi, bioweathering, Salar de Uyuni, quaternary terraces, carbonates

## Abstract

The Chiquini and Galaxias caves contain speleothems that are templated by long fungal structures. They have been associated with the carbonate lacustrine deposits in the margins of the Coipasa and Uyuni Salar basins. During a wetter episode, such carbonates formed at the end of the last glaciation raising the lake level to more than 100 m in the Tauca events (15–12 ky). Such an event flooded the caves that eventually became a cryptic habitat in the lake. The caves show bizarre speleothems framed by large (>1 m) fungal buildings covering the older algal mineralized structures. Although the origin of the caves is not fully understood, the occurrence of two carbonatic units with very distinctive fabric suggests that they formed in two separated humid events. In this regard, the mineralized algal structures, showing the same features as the lacustrine carbonates, likely formed during the Tauca flooding events in the terminal Pleistocene that inundated older caves. The different caves were exposed to the atmosphere after a drop in the lake level that promoted alluvial erosion by <12–10 ky (Ticaña episode) under arid conditions. A last humid episode rising the lake surface 10 m above the Salar level, which was not enough to inundate the caves a second time, drove the formation of the biospeleothems by fungi biomineralization. The abundance and size of the preserved fungal structures suggest that they were sustained by a stable hydrological activity plus a constant organic supply. While nutrients could have been primarily sourced from the vegetal communities that occupied the exhumated lake margins, they might have also been released from the lacustrine carbonatic unit. The combination of hydrology and biological activities were likely determinants for a fast rock dissolution and mineralization ending in the construction of the fungal biospeleothems.

## Introduction

**Figure 1:**
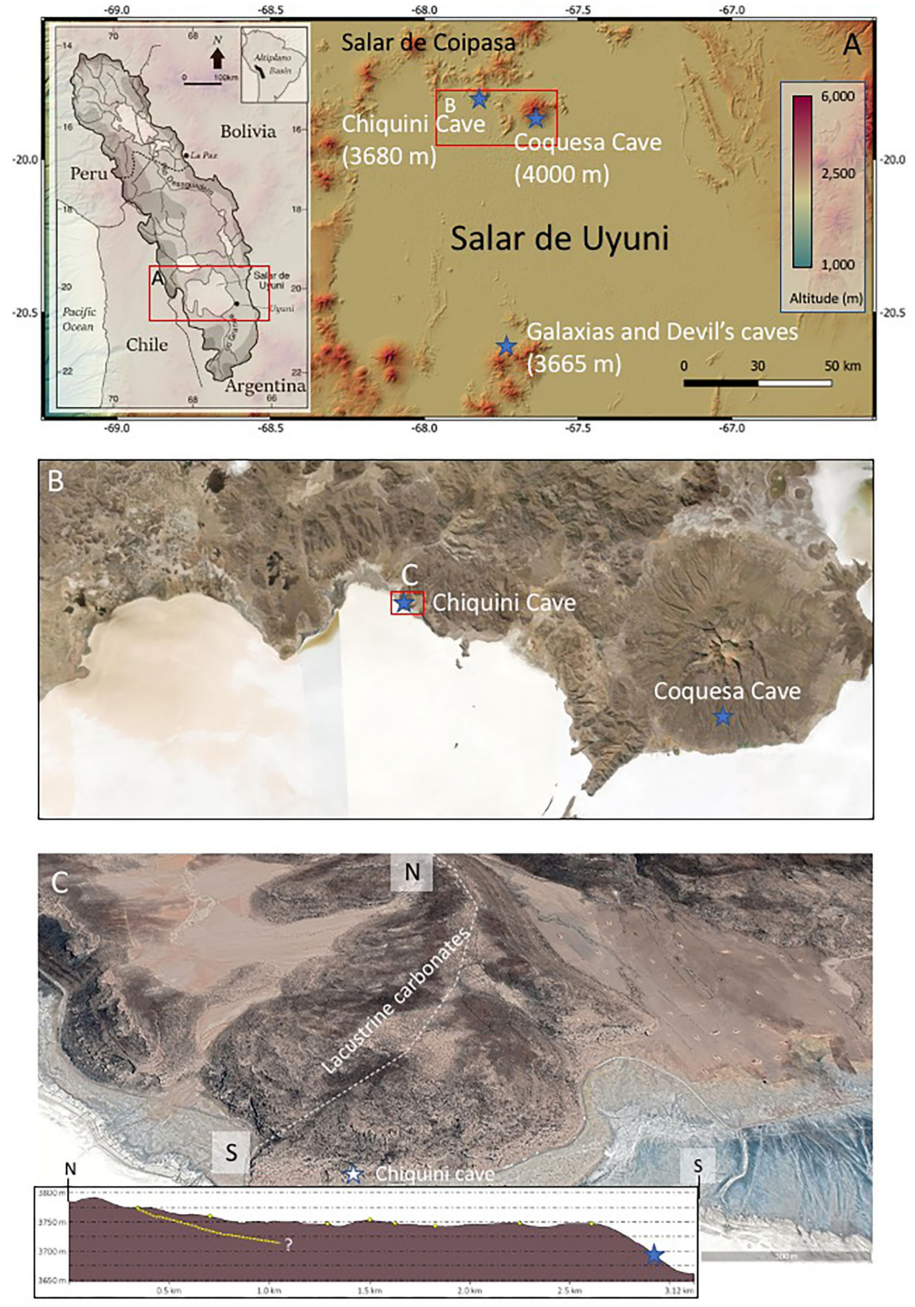
Geographic distribution of the different caves in the Uyuni basin. **(A)** Digital Elevation Model showing the occurrence of the three different caves, including Chiquini, Coquesa, and Galaxias and Devil's. **(B)** Satellite image Situation of Chiquini and Coquesa caves in the North area of the Salar Uyuni. **(C)** Geomorphological settings of the Chiquini cave emplaced in the Pleistocene lacustrine carbonates forming a terrace on the volcanic deposits of Pleistocene age (Tibaldi et al., 2009). The dashed white line traces the direction of the topographic profile, while the yellow dashed line traces the boundary between the volcanic and lacustrine deposits inside the altitude profile.

Along the margins of the Coipasa and Uyuni Salar basins (Figure 1), different caves occur in the lower part of a lacustrine carbonate unit, which is composed of biohermal and algal structures (Servant and Fontes, 1978). Such carbonate deposits formed through a transgressive episode during the terminal stages of the last glaciation under humid conditions by 15–12 ky (Sylvestre et al., 1995, 1999). As a result of this lacustrine transgression, the caves occurring below 3,690 m were completely flooded and partially infilled by mineralized biological structures of calcareous composition. In this regard, mineralized algal fabrics in the interior of the caves similar to the lacustrine biosedimentary fabrics likely record the transgressive episode that formed the carbonate shelf in the lake. Intriguingly, a very distinctive set of speleothems framed with mineralized fungi are also found covering the ceiling and floor of the caves, which are mainly formed by the older cryptic lacustrine carbonates. The occurrence of speleothems in the cave suggests that they were formed during a drop in the lake level under humid conditions that sustained an active weathering of the lacustrine carbonates deposited in the Late Pleistocene (Sylvestre et al., 1995, 1999).

**Figure 2:**
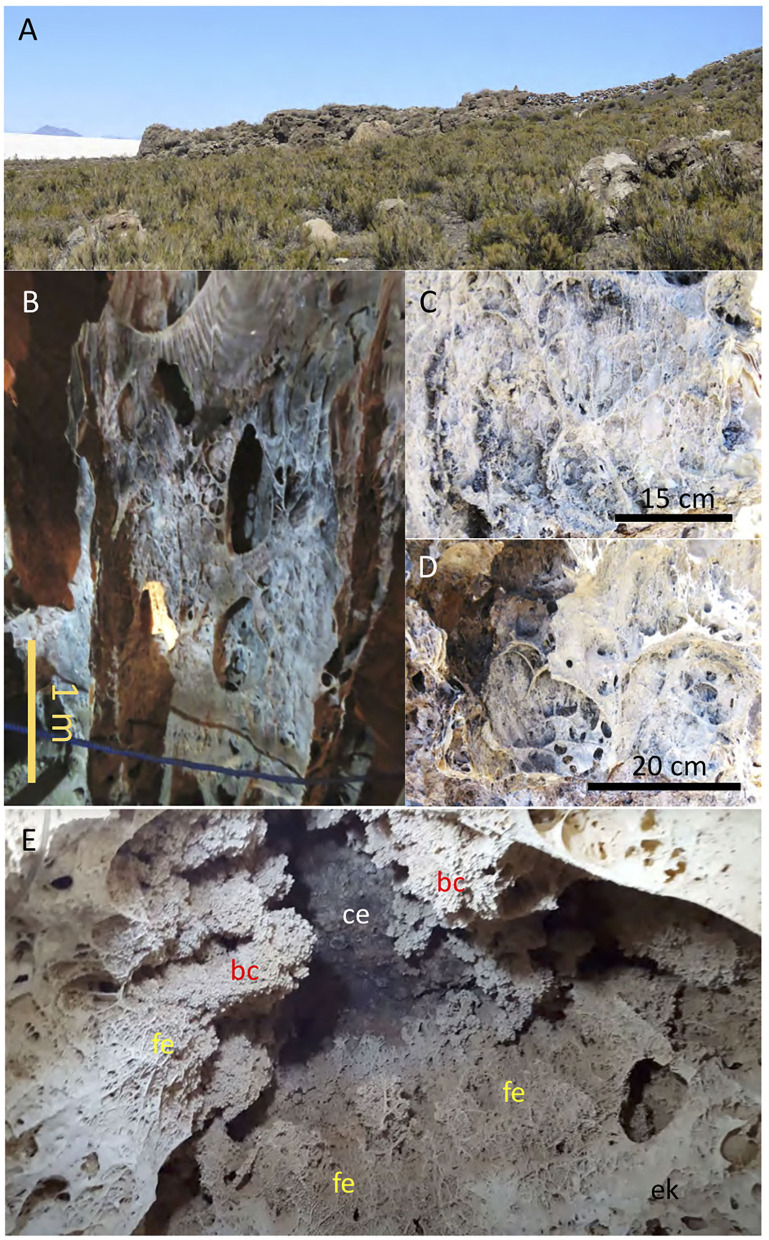
Pictures showing the outcrops of carbonate deposits of lacustrine and cave environments. **(A)** Lacustrine terrace of late Pleistocene carbonates (Rouchy et al., 1996; Sylvestre et al., 1999) around the Chiquini cave. **(B)** Speleothem in Chiquini cave framed by long filaments **(C,D)** mineralized by Mg-rich carbonate. **(E)** Image pointing the Galaxias cave ceiling (ce) of volcanic composition that is covered by biohermal carbonates (bc), which are encased by long and thick mineralized filaments (fe) building the cave speleothems. In such material succession, the biohermal carbonates (bc) formed inside the cave by karstic processes are older than the biohermal structures (bc), which formed when the cave was fully flooded. The picture showing the Galaxias cave ceiling in E is a courtesy of Geoffrey SG.

The speleothems are mineralized by long filamentous structures of microbial fungal hyphae (Figure 2). Although it has been reported that fungi are a common component in the microbial communities in karstic systems and caves (Engel, 2011; Hershey et al., 2018), they are not found as the main mineralizing agent in the construction of the speleothems. However, the occurrence of the Uyuni speleothems framed by a dense network of fungal hyphae (Figure 2) has not been reported on such a large scale. When considering microbial mineralization, most studies concern prokaryotes. However, induced microbial biomineralization by eukaryotes is scarcely documented, especially when considering the fungal kingdom (Sterflinger, 2000; Burford et al., 2003; Gadd, 2008; Gadd and Raven, 2010). Fungi are everywhere on the surface of the Earth, wherever there is oxygen. They are also able to survive without oxygen, however, this is not their natural lifestyle (Bindschedler et al., 2016). The most preferred habitat for fungi is believed to be soil, but they are also very common in rock surfaces and caves (Sterflinger, 2000; Ritz and Young, 2004; Vanderwolf et al., 2013).

Fungi are chemo-organo-heterotrophic organisms, thus, depending on organic matter to survive and sustain their metabolism (Bindschedler et al., 2016). They obtain their carbon source either from associations with living partners or from available organic matter (Bindschedler et al., 2016). Fungi acquire their nutrients by absorption, by, first, pre-digesting their substrate using oxidative or hydrolytic enzymes secreted in the external environment, and then, transporting solubilized nutrients inside their cells. Therefore, their metabolic activity depends mainly on the available nutrients. Fungi also interact with inorganic minerals and metals and are, thus, involved in the biogeochemical cycling of compounds, such as Ca, Fe, K, and Mg (Bindschedler et al., 2016).

In this regard, it is known that fungi also colonize rock surfaces in arid environments, interacting with their mineral substrate, therefore, influencing their physical and chemical stability (Gorbushina, 2007; Parchert et al., 2012). Fungi are also known to be involved in both CaCO_3_ bioweathering and biomineralization (Verrecchia and Dumont, 1996; Sterflinger, 2000; Burford et al., 2006; Kolo et al., 2007; Hou et al., 2013). Both bioweathering and biomineralization are strongly related since the products released from the CaCO_3_ dissolution can further re-precipitate as CaCO_3_ depending on factors, such as pH, temperature, carbonate alkalinity, and pCO_2_ (Burford et al., 2006). Carbonate alkalinity is mainly controlled by the pH level, which in turn will define the carbonate species in the solution, therefore, to precipitate CaCO_3_ two factors are of crucial importance: the carbonate alkalinity and the calcium concentration (Ca^2+^) (Castanier et al., 1999; Dupraz et al., 2009). Fungal activity can influence both of those factors. Additionally, fungi can also influence those two factors through organomineralization (biologically influenced mineralization), as fungal cell walls can adsorb various cations, such as Ca^2+^ (Bindschedler et al., 2016). Numerous past studies on fungi organomineralization prove that this process might be more important than previously thought (Dupraz and Visscher, 2005; Dupraz et al., 2009; Bindschedler et al., 2014).

Consequently, there are various factors in relationship to fungal communities influencing the stability of CaCO_3_. In addition, as fungi are provided by rigid and resistant filaments consisting of elongated cells arranged one after another, they have been observed to actively drill into mineral surfaces (Jongmans et al., 1997; Van Schöll et al., 2008; Moore et al., 2011), and take advantage for the structural Spatio-temporal and nutritional heterogeneities in the rock substrate.

In this article, we investigate the role of fungal communities in forming the speleothems that occur in the caves associated with the carbonates formed during the terminal Pleistocene and early Holocene in the Uyuni Salar. For such a purpose, we will conduct a multidisciplinary approach that linked the area's paleoclimatic evolution with microbial communities' development. The fungal structures are directly involved in the formation of the speleothem buildings but are also an active agent in rock weathering and ion mobilization. Thus, wet climatic conditions found in different episodes of the Pleistocene and the Holocene in the region would have favored weathering and mineralization. In this context, we explored how the climatic activity drove the biogeochemical pathways ending in the formation of the speleothems through the activity of the fungal communities in the Uyuni and Coipasa basins.

## Environmental and Geological Settings

### Regional Settings

The Altiplano of Bolivia is a 200,000 km^2^ internally drained basin, located between the western and eastern of the Andes Cordillera at an altitude of 4,000 m (Figure 1A). The western cordillera has a volcanic origin. Volcanoes, ignimbrites, and lava flow generally overlie the Cenozoic formations by more recent volcanic activity (Risacher and Fritz, 1991; Salisbury et al., 2015). The eastern cordillera contains Paleozoic sediments (shales and sandstones) and granitic plutons, which constitute the Altiplano basement (Risacher and Fritz, 1991). This basement is filled with continental sediments from the Cretaceous and Cenozoic ages. During the Pleistocene, the central and southern part of the Altiplano was persistently covered by large saline lakes, characterized by alternating episodes of expansion and desiccation, a phenomenon commonly explained by climatic fluctuations in the region (Rouchy et al., 1996; Fornari et al., 2001). The depth and extent of these large paleolakes varied greatly depending on variations in the rainfall/evaporation rates (Hastenrath and Kutzbach, 1985; Servant et al., 1995; Sylvestre et al., 1995). Well-preserved outcrops are recorded from the last two of these lacustrine phases, the Minchin (30,000–20,000 yrs) and Tauca (>16,000–14,000 yrs) episodes (Sylvestre et al., 1999; Fornari et al., 2001). A development of carbonate accumulations took place during successive lacustrine highlands, discontinuously covering the terraces and slopes of the paleolake (Rouchy et al., 1996; Placzek et al., 2006). During lowstands, the level of the lake dropped, leaving behind salt deposits in the deepest part of the basin, which corresponds to the current Salar de Uyuni (Rouchy et al., 1996; Sylvestre et al., 1999; Fornari et al., 2001).

### Geological and Geobiological Settings of Caves

The basement of the Uyuni and Coipasa basins is formed by a large synclinal structure (Corque syncline) composed of thick (>10 km) synorogenic deposits of Eocene to Oligocene age (Mcquarrie, 2002). The basins are limited by younger volcanic edifices and lava flows (Figure 1B) dating back to the late Cenozoic and the Early Quaternary (Tibaldi et al., 2009; Salisbury et al., 2015). Such materials were subsequently reworked by fluvioglacial activity that has been recorded as moraines, which are found covering the Tunupa hillsides above 4,100 msl (Clapperton et al., 1997). During the Pleistocene, the synclinal structure was flooded by different water bodies, which formed through different humid episodes (Sylvestre et al., 1999; Argollo and Mourguiart, 2000). The water level was high enough to join the Uyuni and Coipasa Salars in the gigantic paleolake, known as Tauca, dating back to 120 ky (Martin Léo et al., 2018). The paleolake reached a maximum level of ~ 3,760 msl at 40 and 16 ky in the area of study (Figures 1B,C) during the Minchin and Tauca phases (Sylvestre et al., 1999; Chepstow-Lusty et al., 2005), respectively. The highstand paleolake episodes in the area hosted the formation of carbonate deposits, which mineralized very diverse structures of biological origin (Rouchy et al., 1996; Blard et al., 2011).

In the Uyuni Salar, caves are found associated with volcanic materials (Figures 1A–C). There is little scientific information about the cave's origin in the area. Thus, the information comes from the inspection of caves done in the Chiquini and Coquesa locations (Figures 1B,C) suggesting that are emplaced in volcanic materials. The Coquesa cave formed in breccias and conglomerates of the Tunupa volcano, which shelter several mummies of the Chullpa people. This cave shows no evidence of karstic structures as occurs at ~4,000 m well above the different highstand episodes inundating the Uyuni and Coipasa basins (Sylvestre et al., 1999). On the contrary, the Chiquini cave, occurring at ~3,680 m, is found below the carbonate unit (Figures 1C, 2A), formed during the Tauca phase in the terminal Pleistocene (Sylvestre et al., 1999). In this case, the cave is filled with abundant speleothems framed by long buckles of hanging filamentous structures from the cave ceiling to the floor (Figures 2B–D). Chiquini cave measures up to 5 m high and more than 10-m width. The speleothems are, in the majority of cases, of high magnitude, as they can be up to 4-m high. Some of them reach the cave floor and seem to be associated with stalagmites. We believe the speleothem formation is associated with a humid episode that incremented the lake dimensions, suggesting that the speleothems were formed by water circulation.

Interestingly, the same structures are found in the Gruta de las Galaxias (Cavern of the Galaxies). Such a cave occurs 4 km west of the Aguaquisa village at ~3,670 m (Figure 2E) flanked by alignments of Miocene volcanic edifices (Tibaldi et al., 2009), like Caltama and Qaral. The Cavern of the Galaxies also occurs below the carbonatic deposits of the Tauca phase. It hosts the same structures framed by networks of long filaments, which grow from a volcanic ceiling covered by carbonates showing a different fabric (Figure 2E). Such a fabric consists of short-branched stems that are also found in the thrombolytic structures of the Tauca lacustrine carbonates (Rouchy et al., 1996; Blard et al., 2011). The presence of two different carbonate materials in the interior of the caves suggests that they were formed under different environmental conditions. While the speleothem carbonates formed in a karstic system, the ceiling's thrombolytic carbonates were likely formed when the cave was completely flooded during a highstand episode in the area (Sylvestre et al., 1999).

## Methods

### Sample Collection

A geological survey was performed to inspect the lacustrine carbonate terraces and caves in the Uyuni Salar (Figures 1C, 2A–D, Supplementary Figures 1, 2, Supplementary Table 1). We mostly looked for the different carbonate structures to distinguish the biospeleothems from the lacustrine materials in the interior of caves. Samples 134–1, 134–2, and 134–4 were collected from two different speleothems in the Chiquini cave to characterize the speleothem's geobiological content through the microstructure and elemental composition under the Scanning Electron Microscope and Electron Dispersive Spectroscopy (SEM-EDS). In addition, lipid analyses were conducted to determine the speleothem biological origin (Supplementary Table 2). Some additional samples like 129, from the lacustrine carbonates, and 134–3, obtained in the outcrops occurring at the cave entrance, were also collected to compare their structure, elemental, and molecular composition with the speleothem carbonates. It was done to recognize differences in the biological communities as the lacustrine carbonates formed under quite different environmental conditions than the speleothems, which should be accordingly observed in the molecular record of organic compounds and biomolecules. Samples were carefully collected using nitrile gloves, covered by aluminum foil, and introduced into a sterilized sampling bag to prevent contamination.

### Mineral Identification by X-Ray Diffraction

The mineral identification of the carbonate samples 129, 134–1, 134–2, and 134–3 (Supplementary Table 1) was done through the X-ray diffraction technique. For such a purpose, a fragment of the speleothem sample was powdered using an agate mortar. The mineral characterization was done by a Seifert 3003 T–T X-ray diffractometer (copper radiation source) scanning 2θ° diffraction angles from 0 to 70°. For mineral identification, we performed a semi-quantitative analysis using the RIR (Reference Intensity Ratio) method from the I/Ic data (intensity ratio of the highest intensity peak of the phase, compared to the most intense corundum peak). This value is tabulated for many of the phases in the database that we have. When the identified phase has no value for I/Ic, the value of 1 is automatically assigned. The calculation of the semi-quantitative analysis assumes that all the phases are identified, so that the software assumes that the sum is 100% (*Σci*= 100%). The mineral identification was performed using the Diffract.Ev, a program under the PDF2 mineral database.

### Scanning Electron Microscope and Electron Dispersive Spectroscopy

Microscopic and chemical evaluation of the Uyuni samples was performed by three types of Scanning Electron Microscopes, including: (1) a JEOL-5600, coupled to an Oxford INCA X-sight EDAX Energy Dispersive X-ray Microanalysis, (2) a JEOL IT500 coupled with an Oxford MAX170 microanalysis, and (3) a Scanning Electron Microscopy-Field Emission Gun (SEM-FEG) Philips XL30-FEG. SEM measurements and chemical analyses (EDS) were performed on uncoated and gold-coated sample pieces using a ZEISS EVO 10 (Carl Zeiss, Oberkochen, Germany). Before analysis, the sample was repeatedly cleaned with a rubber air dust blower to eliminate impurities. Electrically conductive carbon tabs and double sticks were pressed to conductive graphite stubs and were gold-coated using a Quorum, Q150T-S device to enhance electrical conductivity and prevent charging under electron beams. Various stubs with sample pieces were then placed inside the SEM chamber in high vacuum mode to analyze the sample microstructure with a secondary electron detector. The Philips XL30-FEG was used to perform SEM-EDS analysis to follow the microstructure and composition variation of samples showing a diverse structure like the 134–3, which is a heterogeneous material displaying a varying structure. Analytical conditions were variable set at 0.2 mA current and 15 kV accelerating voltage for the uncoated samples, while conditions for the gold-coated samples were 50 pA and 10 kV.

### Transmission Electron Microscope

The TEM was used to reveal the internal microstructure of sample 134–1 corresponding with a speleothem fragment with laminated microstructure. For such a purpose, the sample was consolidated and fixed in 4% paraformaldehyde and 2% glutaraldehyde in 0.1-M phosphate buffer (pH 7.2) for 2 h at room temperature. The fixed samples were subsequently washed three times by the phosphate buffer and post-fixed with 1% of OsO4 in water for 60 min at room temperature in the dark. Later, they were washed three times by distillate water, and subsequently incubated with 2% aqueous uranyl acetate for 1 h at room temperature, washed again, and dehydrated in increasing concentrations of ethanol 30, 50, and 70% at 20 min each, 90% 2 × 20 min, and 100% 2 × 30 min at room temperature. Dehydration was terminated with a mixture of ethanol/propylene oxide (1:1) for 10 min and pure propylene oxide for 3 × 10 min. Infiltration of the resin was accomplished with propylene oxide/ Epon (1:1) for 45 min and pure LR White resin (London Resin Company limited, England), overnight at room temperature. Polymerization of infiltrated samples was done at 60°C for 2 days. Ultrathin sections of the samples were done using an Ultracut of Leica that was stained with uranyl acetate and lead citrate by standard procedures.

### Extraction and Analysis of Lipid Biomarkers

We performed the analysis of the total lipid extract (TLE) to identify the main biological groups that have been involved in the carbonate formation in the lacustrine and cave paleoenvironments. Lipids of four lyophilized and ground subsamples (50–80 g) of the Uyuni speleothems were extracted with ultrasound sonication (3 × 15 min) using 15 ml of a 3:1 (v/v) mixture of dichloromethane (DCM) and methanol (MeOH) to obtain ca. with 45 ml of TLE. Before the extraction, tetracosane-D_50_, myristic acid-D_27_, and 2-hexadecanol were added as internal standards.

The concentrated and desulfurized TLE (Sánchez-García et al., 2018) was hydrolyzed overnight with KOH (6% MeOH) at room temperature (Grimalt et al., 1992). Then, liquid-liquid extraction with *n*-hexane (3 x 30 ml) was performed to recover the neutral fraction first and the acid compounds afterward, after acidification with HCl (37%) (Sánchez-García et al., 2020). Further separation of the neutral fraction into non-polar (hydrocarbons) and polar (alkanols and sterols) was done according to a method described elsewhere (Carrizo et al., 2019). The acidic fraction was transesterified with BF_3_ in MeOH to produce fatty acid methyl esters (FAME), and the polar fraction was derivatized with N,O -bis [tri- methylsilyl] trifluoroacetamide (BSTFA) to analyze the resulting trimethyl silylated alcohols (Sánchez-García et al., 2020).

All fractions were analyzed by gas chromatography-mass spectrometry (GC–MS) using a 6850 GC System coupled to a 5975C VL MSD Triple-Axis detector (Agilent Technologies, Santa Clara, CA, USA), which operated with electron ionization at 70 eV and scanning from m/z 50 to 650 (analytical details can be found in Sánchez-García et al., 2020). Compound identification was based on retention time and mass spectra comparison with reference materials and the NIST mass spectral database. Quantification was performed with the use of external calibration curves of *n*-alkanes (C_10_-C_40_), FAME (C_6_-C_24_), and *n*-alkanols (C_14_, C_1,8_, and C_22_), all supplied by Sigma-Aldrich (Madrid, Spain). A procedural blank doped with the three internal standards were analyzed in parallel to the samples, to check for contamination and estimate the method recovery (74 ± 16%). No significant contamination of target analytes was recorded. Lipid concentration was measured as micrograms per gram of dry weight sample (μg·gdw^−1^).

## Results

### Cave Inspection

In July 2019, we visited and sampled the Chiquini and Coquesa caves that are emplaced in the volcanic deposits of the Pliocene age (Tibaldi et al., 2009) produced by the activity of the Tunupa volcano (Figure 1A). As discussed above, the Coquesa cave occurs at ~4,000 msl showed no evidence of any speleothem material but was formed by brecciated deposits of volcanic composition. On the other hand, the Chiquini cave (Figures 1A–C) emplaced at ~3,670 msl under a lacustrine carbonate terrace (Figure 1A) is occupied by a dense population of speleothems that are framed by large filaments (Figures 2B–D). The cave ceiling is covered by two different carbonate deposits (Figure 2E), which are distinguished by their distinctive fabrics. The first unit consists of carbonatic deposits with millimeter- to centimeter-long short-branched structures, which occur at the cave ceiling following a patchy distribution. They are encrusted and covered by the speleothems buildings that grow from the ceiling to the floor cave. They are composed of networks of long-branched filaments (>10 cm), whose filament density decreases, but their length increases from the ceiling to the cave bottom (Figures 1B, 2E). As the building growth is controlled by the development of filamentous networks (Figures 2B–D), they usually have a flat morphology, whose size is limited by the ceiling height and the fungal network growth. As a result, they form flattened speleothems with an internal complex structure merging different corrugated and undulate surfaces (Figures 2A–D). The same distribution of carbonate materials is also observed in the Galaxias cave (Cueva de las Galaxias) found NW of the Colcha location at an altitude of 3,665 msl (Figures 1A, 2E). In turn, the Galaxias cavern is more densely populated by filamentous buildings than the Chiquini cave, which show a larger volume and a more complex structure formed by corrugated laminae. Interestingly, the Chiquini and Galaxias caves do not host any hydrological activity associated with the speleothem formation, which agrees with the current arid climate.

### Mineral Identification

The XRD analysis of different samples collected in the lacustrine carbonate deposits and the Chiquini cave has resulted in identifying different calcite type minerals characterized by the magnesium concentration (Supplementary Figures 4A–C, Supplementary Table 1). In this regard, the XRD analysis from the lacustrine carbonates with columnar fabrics in Coipasa (sample 129) results in low magnesium calcite (LMC), which diffraction diagram matches a composition of Mg_0.03_Ca_0.97_CO_3_ in the PDF2 database (Supplementary Figure 3A). Samples 134-1 and 134-2, corresponding with the speleothem laminated carbonates (Supplementary Figure 3B, Supplementary Table 1), have provided a mineral composition that also fits well in an LMC crystal structure (Mg_0.06_Ca_0.94_CO_3_). Furthermore, the XRD analysis shows that the carbonatic tuff (sample 134–3) is mostly composed of high magnesium calcite (HMC) (Mg_0.1_Ca_0.9_CO_3_), which comes together with a secondary carbonate identified as kutnahorite [Ca_1.11_Mn_0.89_(CO_3_)_2_] (Supplementary Figure 3C).

### SEM-EDS Analysis

**Figure 3:**
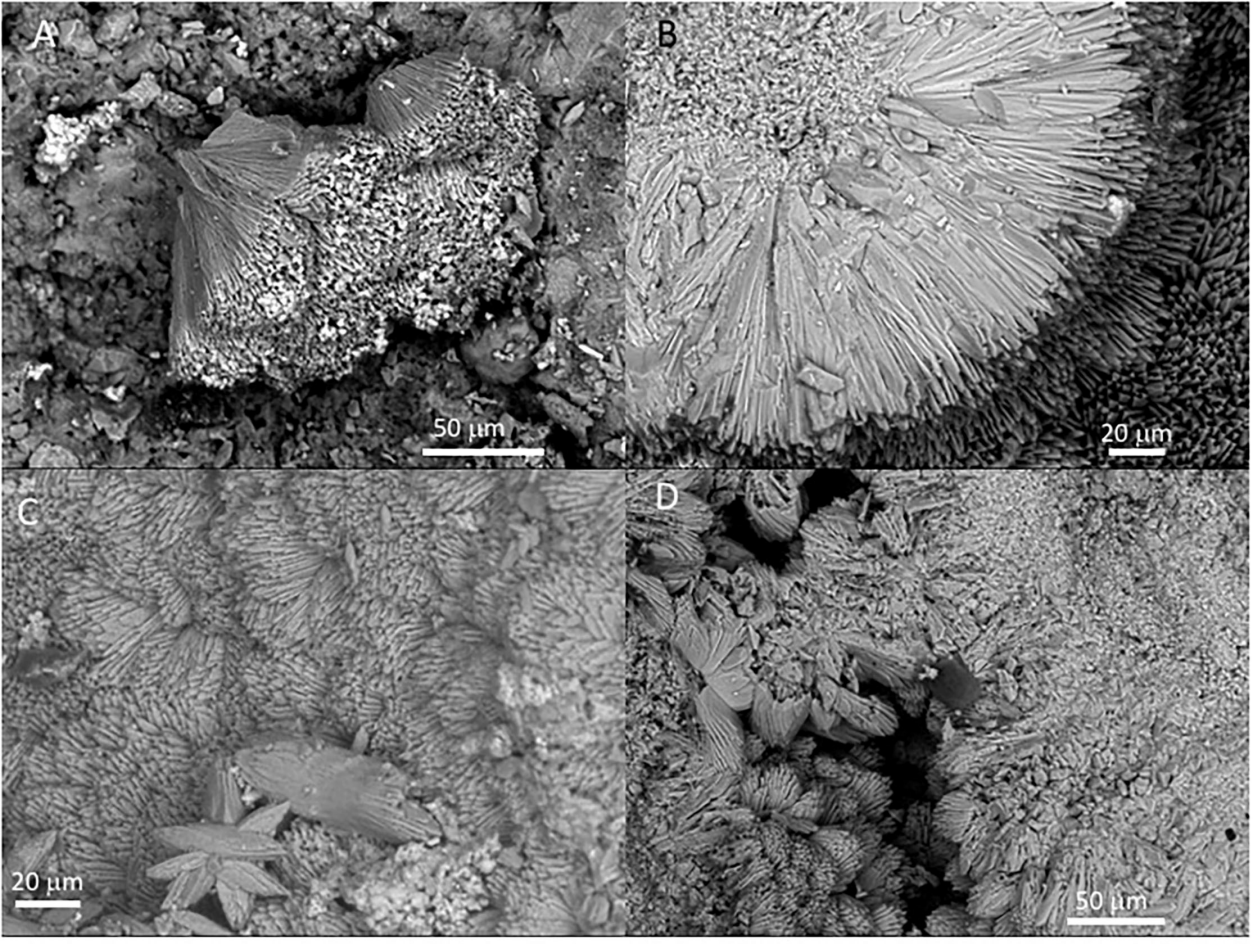
Scanning Electron Microscope (SEM) of Mg-calcite crystals occurring at the outer sheet in the speleothem sample 134–4. **(A)** Fan-like intergrowth of Mg-calcite needles. **(B)** Detail of pristine primary mineralogy of calcite with needle-like crystals. **(C)** Star-shaped microstructures from the aggregation of calcite needles. **(D)** Coalescence of two external sheets with a needle-like microstructure.

**Figure 4:**
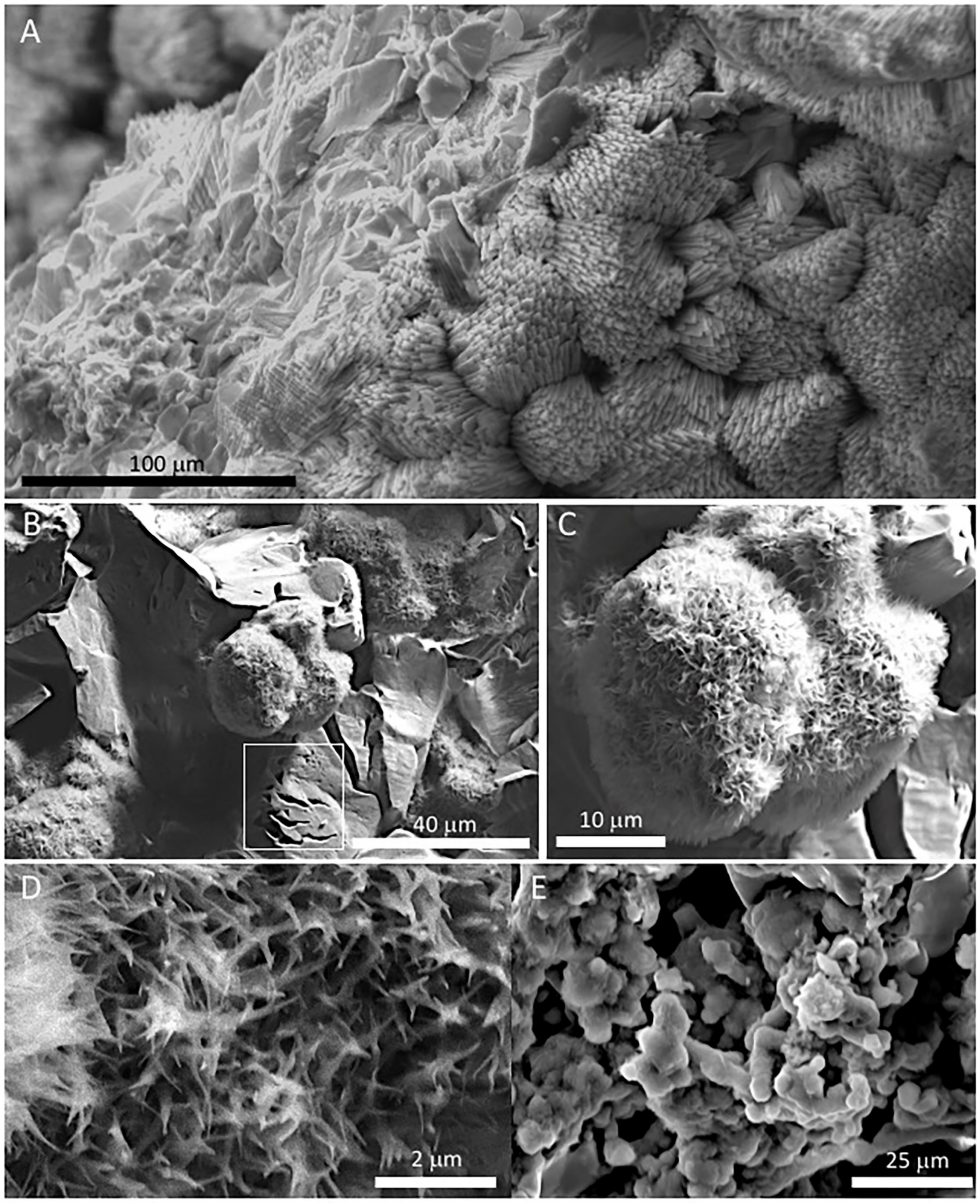
SEM images of samples 134–1 show the massive inner lamina **(A)** occurring beneath the 10-micron thick needle-bearing sheet. It has microporosity filled by different mineral microstructures as ovoidal to laminar elements **(B,C)** that are formed by **(D)** a spicule-like mesh; and, additionally, by **(E)** aggregates of micron-sized ovoid- to rod-like units. The white square in **(B)** marks the presence of microborings having a diameter lower than 5 microns. The SEM image in **(A)** suggests that the massive microstructure of the inner lamina results from the tight packing of the same needle crystals found in the external sheet.

**Figure 5:**
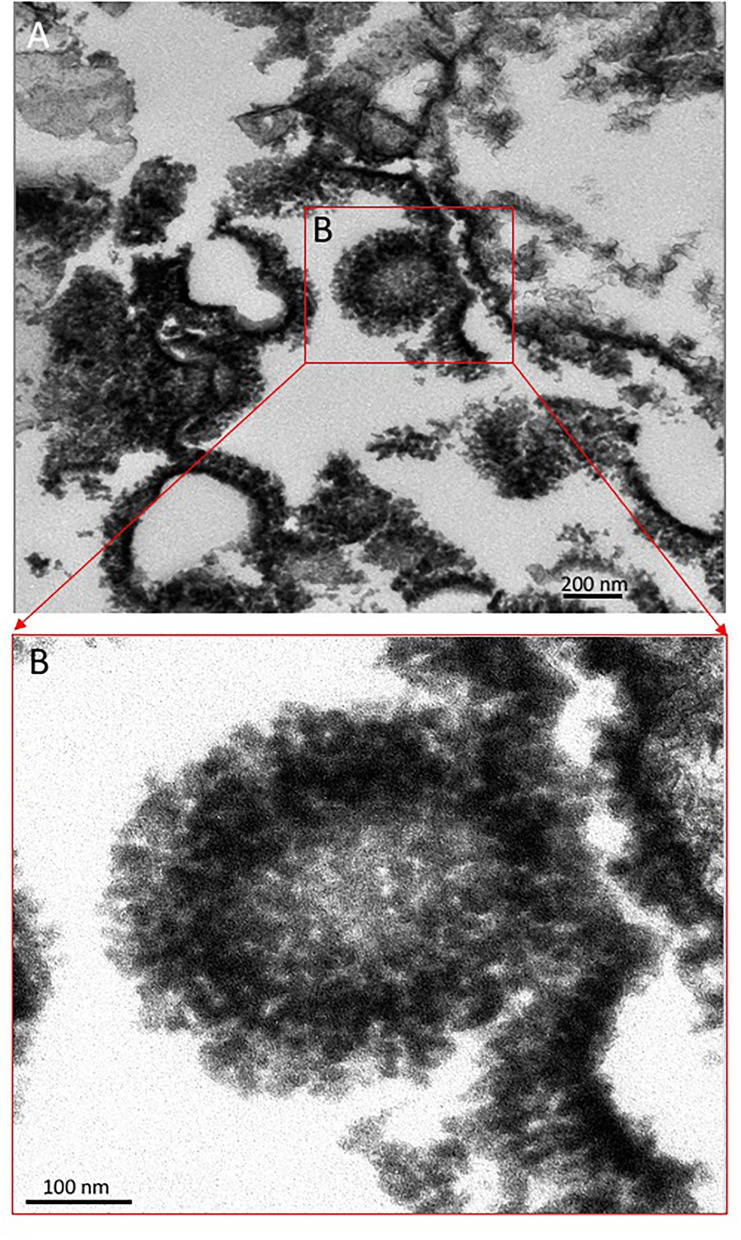
Transmission Electron Microscope (TEM) image from sample 134–1 **(A)** showing the internal structure of the spicule-meshed elements displaying sheet to ovoidal morphologies that are internally voided. **(B)** Detail of an ovoidal element enrooted in a laminar mesh of spicule-like crystals.

**Figure 6:**
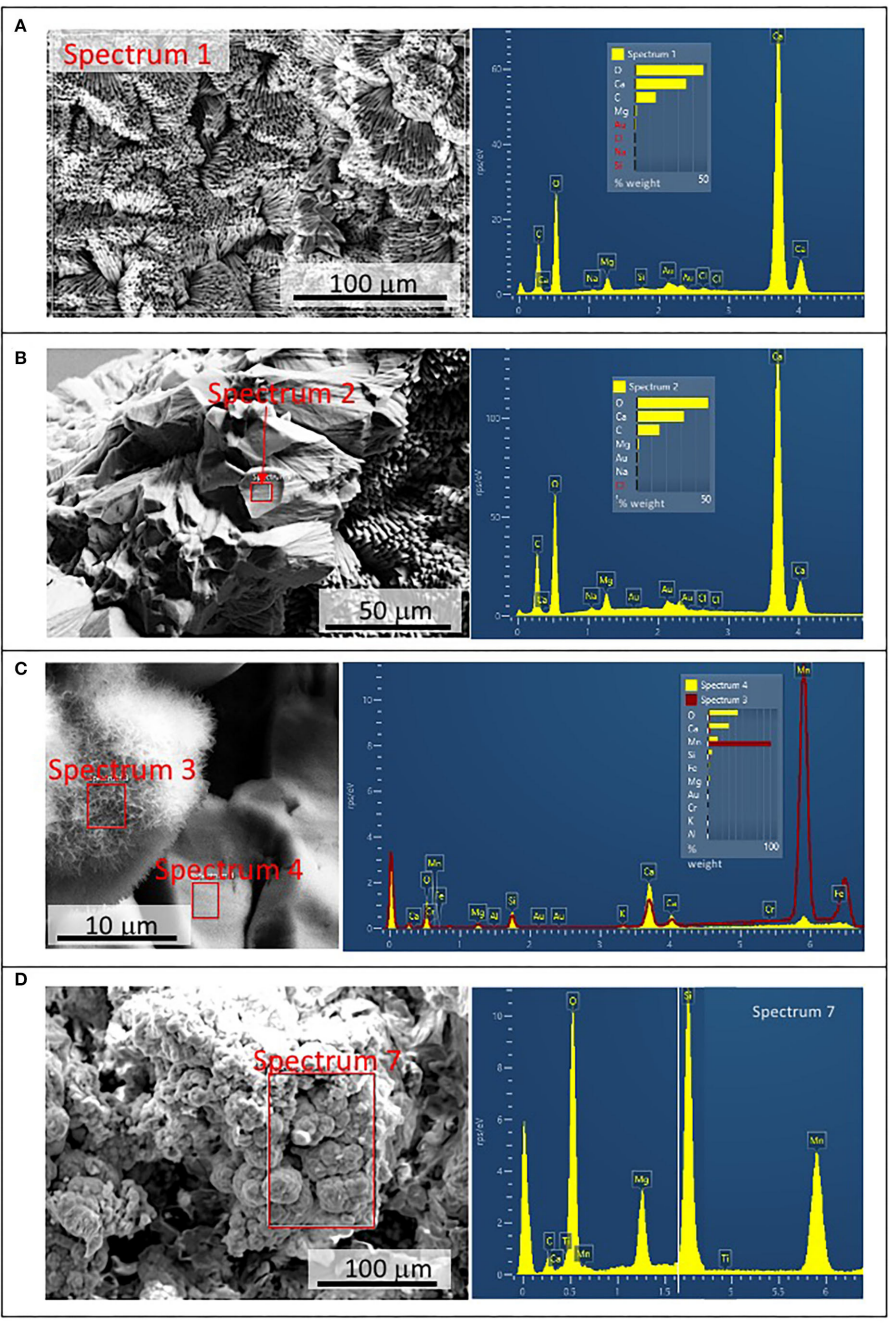
Scanning Electron Microscope and Electron Dispersive Spectroscopy (SEM-EDS) microanalysis of different components found in samples 134–1. **(A)** Chemical composition of the external needle-bearing sheet showing C, O, Ca, and Mg as major elements. **(B)** Microanalysis from the internal layer with the same chemical composition as in the external sheet shown in **(A)**. **(C)** Chemical analysis from an ovoidal element with spicular mesh exhibiting a primary composition of O, Mn, and Fe (spectrum 3 in red), which is very different of the composition of the internal layer (spectrum 4 in yellow) dominated by C, O, Ca, and Mg. **(D)** Microanalysis of the silica-rich aggregates of micron-sized components revealing a high concentration in Si, O, and Mn.

The SEM-EDS analysis of samples 134–1 and 134–4 (Supplementary Table 1) has revealed the occurrence of three different mineral microstructures in the speleothem. They correspond with thin external sheets formed by needle-like crystal palisades (Figures 3A–D, 4A), laminas with a non-crystalline massive microstructure containing discontinuous layers (Figures 4A,B), and spongy globules, whose microstructure is built by nanoscale spicules (Figures 3–**6**).

The external sheet palisades have a thickness of around 2 microns and are composed of 10-micron long and 5-micron thick crystal prisms forming fans, wherein its apex follows an orientation inward of the main structure (Figures 3A–C). The crystal needles can combine to build larger needle-like rays to form star-shaped microstructures (Figures 3C,D). The calcite crystals do not show evidence of secondary mineral alteration, such as recrystallization and corrosion. The sheet composed of needle palisades covers a thicker lamina that has no morphological features, but a massive appearance (Figures 4A,B). It is affected by microporosity filled by other mineral components, like ovoid, to undulated laminas built by a spicular mesh and aggregates of micron-sized and ovoid- to rod-like units (Figures 4C,D, 5A,B). In this regard, the SEM-EDS microanalysis has revealed that the external sheet built with needle palisades and the internal massive lamina is primarily composed of C, O, Ca, and, to a minor extent, Mg (Figures 6A,B). In turn, the spongy undulated to ovoidal microstructures displays a high concentration of Mn and O, while the rod aggregates are mainly composed of Si and O, with minor amounts of Mn (Figures 6C,D).

**Figure 7:**
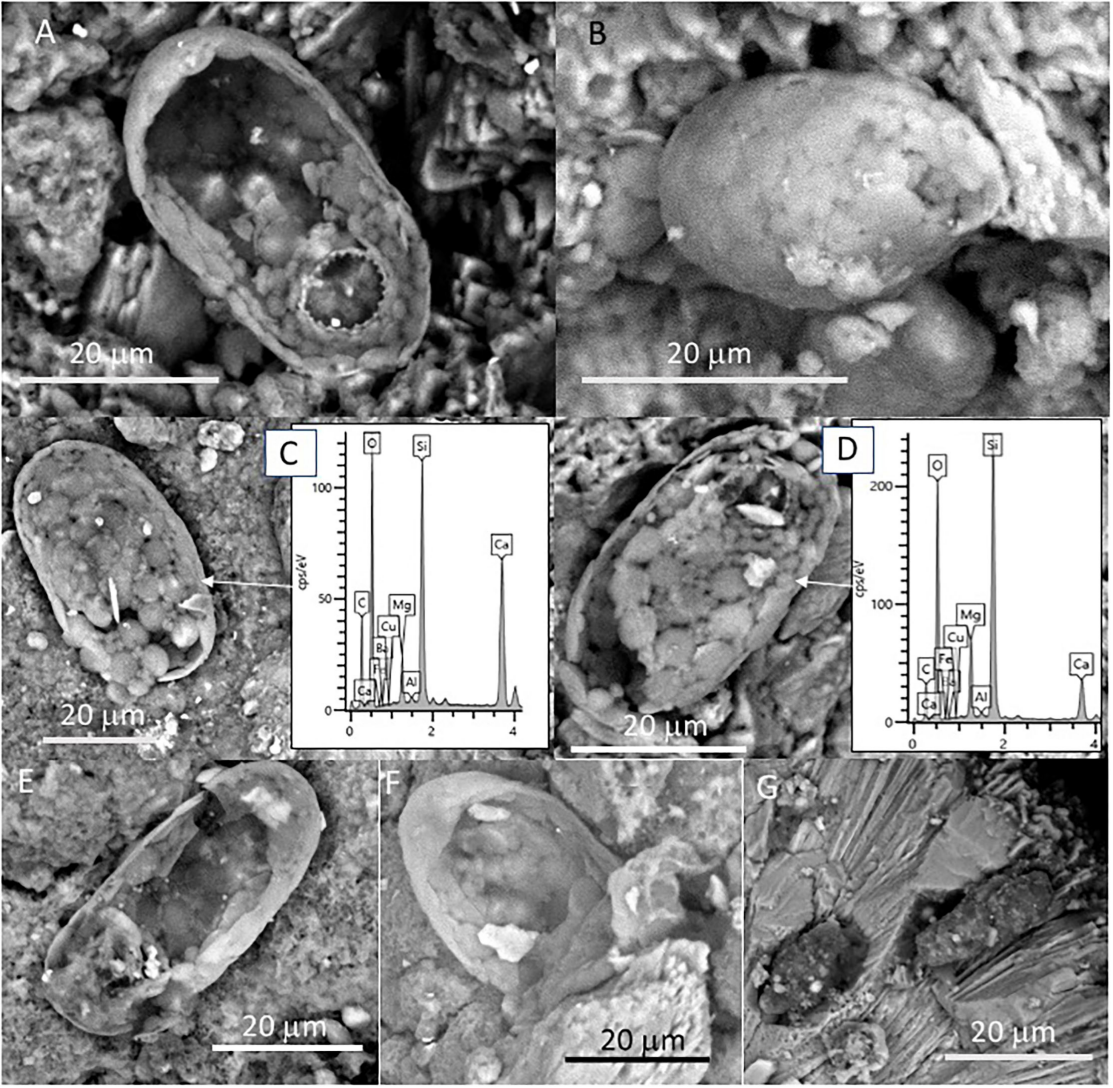
SEM micrographs showing oval-shaped microstructures showing **(A–G)** different preservation stages in samples 134–4. The morphological and compositional features of the specimens suggest that they correspond with the remains of silica-biomineralized testate protozoa. **(A)** Specimen with a dentate operculum. **(B)** Well-preserved specimen revealing the test as the coalescence of different platelets known as idiosomes. SEM-EDS analyses of different specimens **(C,D)** show that the specimen test is composed mainly of Si, C, and O with minor amounts of Mg and Fe, where the Ca likely comes from the mineral matrix. Most of the specimens show varying disintegration degrees of the test **(E,F)** through the degradation of the organic cement releasing idiosomes and ending in the test collapse. Some elements embedded in the mineral matrix **(G)** could correspond with testate amoeba boring the mineral matrix, as they show similar morphological and compositional features.

We have also observed several sack-shaped oval microstructures averaging ~50 microns in size (Figures 7A–G), which, in some cases, have a dentate operculum with circular morphology (Figure 7A). Although most specimens show a partial collapse of the entire structure (Figures 7A,C,D), the complete individuals have a rounded section. The microstructures are built by the amalgamation of circular to elliptical tiny platelets sizing between 1 and 5 mm, which show a composition rich in Si, O, and Mg (Figures 7C,D). In some cases, the platelets are covered by a thin layer of ~2 microns devoid of any evidence of internal fabric and texture (Figures 7E,F). Interestingly, oval microstructures are found embedded inside the crystalline matrix, in which elongation axes are parallel to the needles forming fan bundles (Figure 7G).

**Figure 8:**
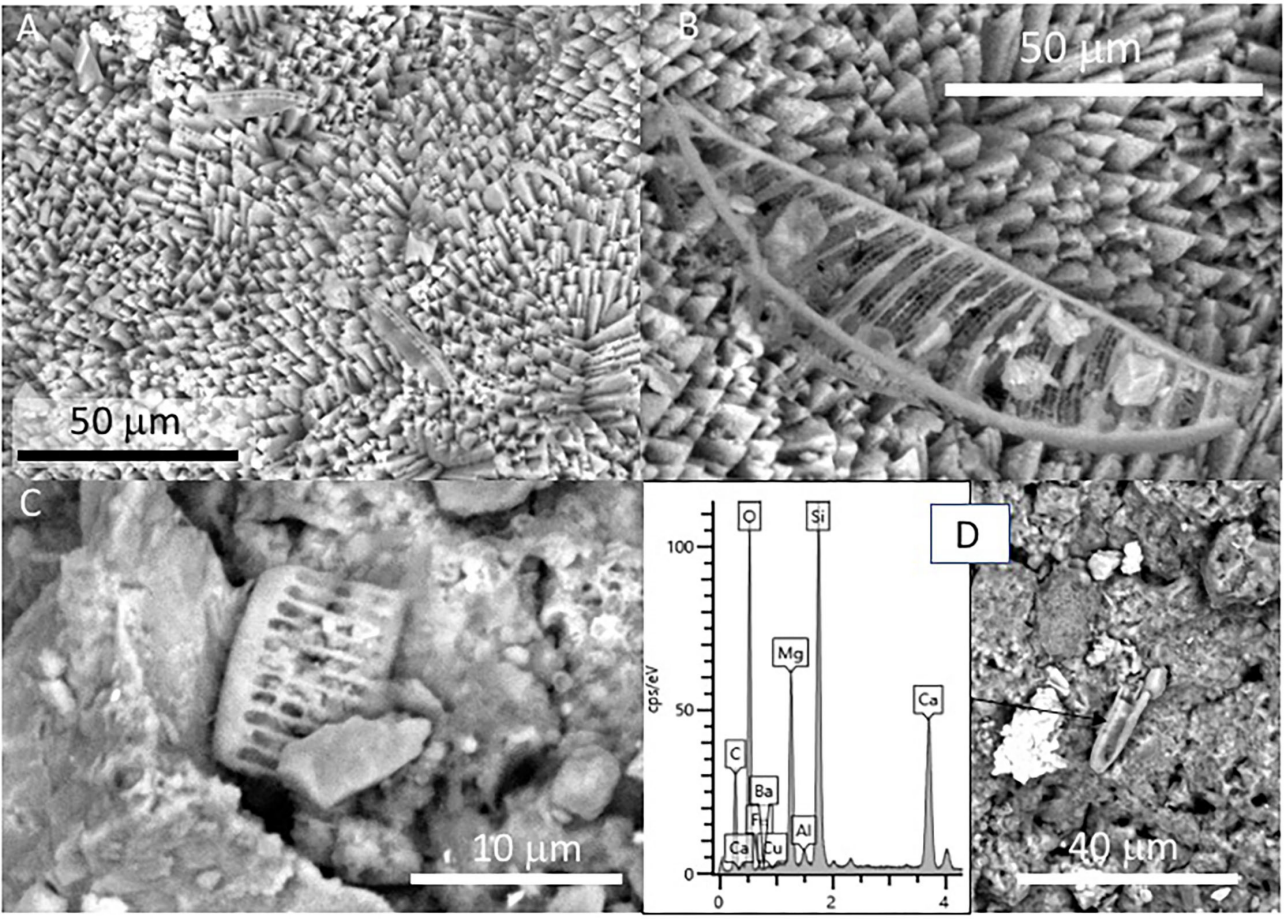
SEM micrographs showing diatom frustules of sample 134–4 collected from a speleothem. **(A)** Two specimens of pennate diatoms located on the surface of the external sheet. **(B)** Detail of one of the pennate diatoms found in **(A)** showing a simple asymmetric valve. **(C)** Frustule fragment of diatom showing a more symmetric morphology included in the internal lamina of the speleothem sample. **(D)** Chemical microanalysis of a diatom frustule characterized by high Si-content together with O, while C, Mg, and Ca is received from the mineral matrix analysis.

Additionally, the SEM analysis of the speleothem sample revealed the presence of shells sizing up to 100μm, with elliptical to round morphology, which showed internal transverse stripes with a varying length of 5 to 20 microns (Figures 8A–D). The EDS microanalysis of such microbial structures shows that it has a high concentration of silicon, oxygen, and magnesium (Figure 8D). The silica-bearing thecae show a very diverse distribution in the mineral substrate as they are found in mineral surfaces, pore spaces, narrow fissures, and cracks on the calcite crystals (Figures 8A–D).

**Figure 9:**
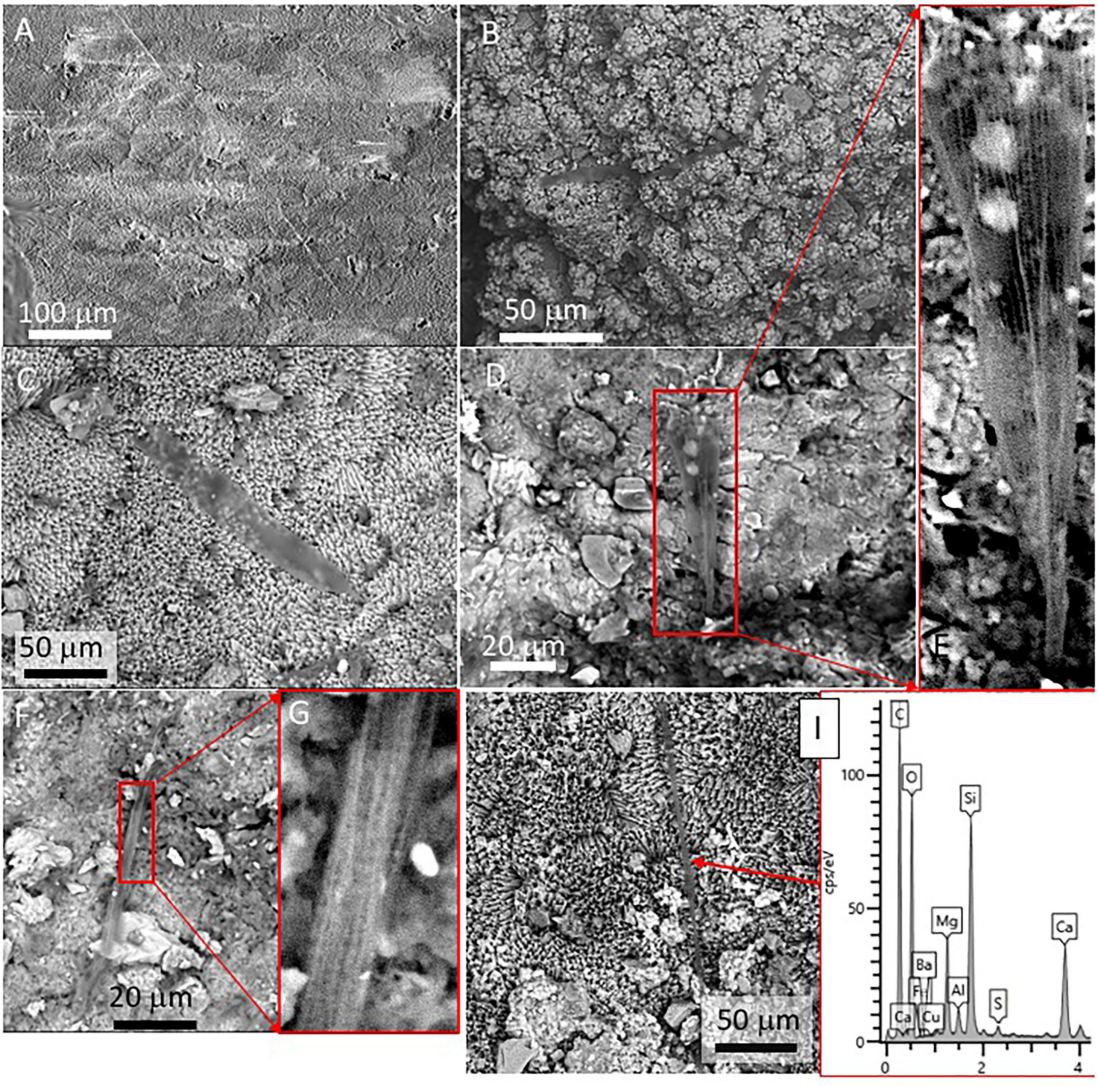
SEM view of thick filaments (10 > microns) in samples 134–4. **(A)** 500-micron long filament, partially mineralized. **(B)** Network of large filaments with varying directions embedded in the mineral substrate. **(C)** Thirty-micron thick filamentous structure showing an apical area attached to the mineral substrate. **(D,E)** Thick filament also with an apical end bond to the mineral surface, which shows € a wall texture consisting of longitudinal and subparallel lines. **(F)** ~5-micron thick straight filament with **(G)** reticulate texture. **(H)** SEM-EDS analysis of a straight filament revealing a high C and Si content with smaller amounts of S suggesting organic composition exposed to silicification. **(I)** Chemical microanalysis of a thick filament, characterized by high C, O and Si content, while Mg and Ca are received from the mineral matrix.

**Figure 10:**
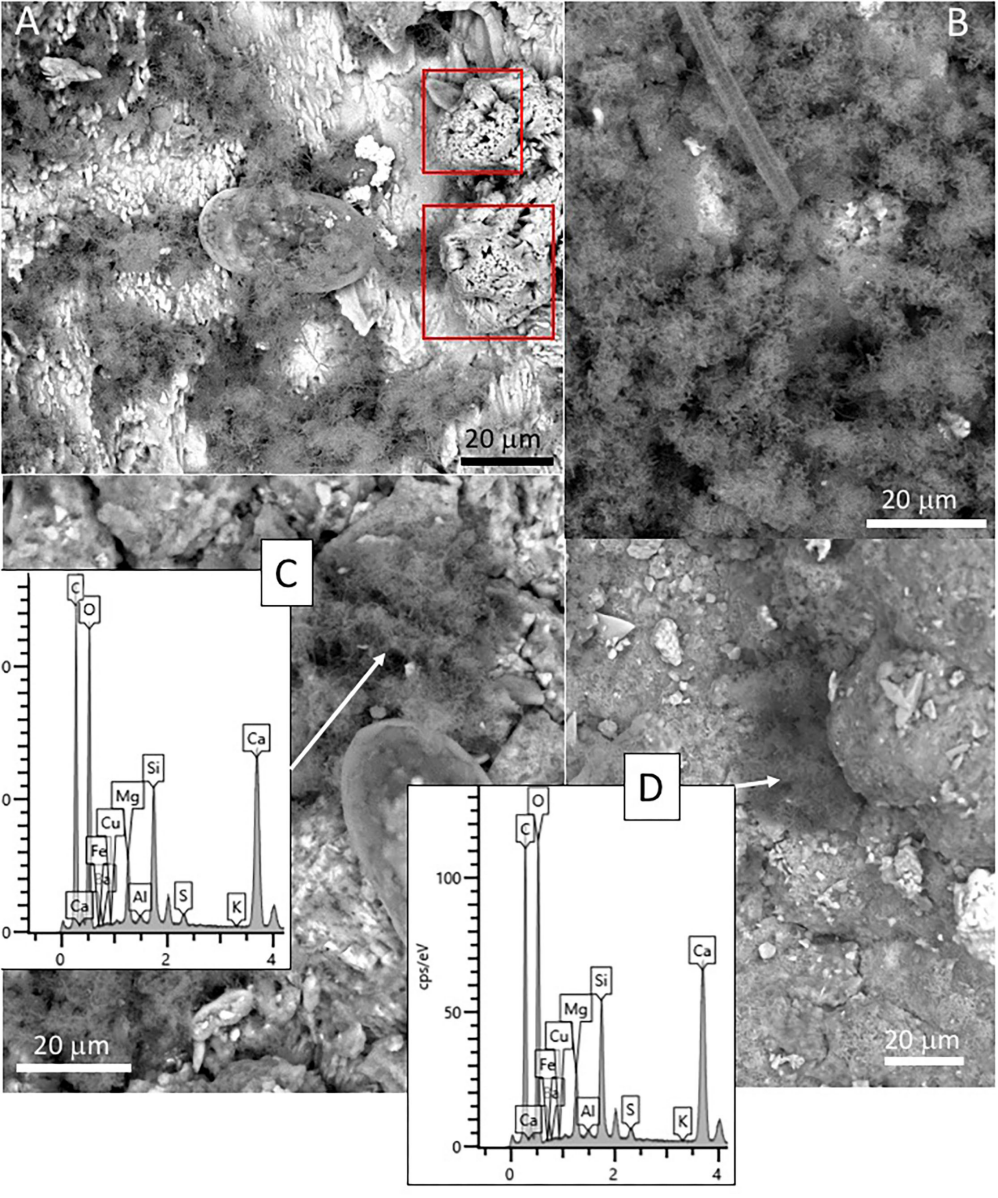
Different images displaying unregular networks of entangled threads interpreted as extracellular polymeric substances (EPS) produced by microbial biofilms. **(A)** EPS embedding one testate amoeba, where red squares are tracing mineral sites with strong corrosion likely of microbial origin. **(B)** EPS densely covering the mineral surface associated with a large filament with reticulated texture. **(C,D)** SEM-EDS analysis of two different sites with EPS revealing a composition dominated by C, Si, and S. Other elements like Ca, Mg, Fe, Al, and Ba are likely sourced on the mineral matrix.

Detailed observations of the sample 134–4 also show several types of filaments with morphologies were observed, which can be grouped into two basic categories: thick (>10 microns), straight to sinuous, and long filaments (Figures 9A–F), and thin (<1 micron), curved, and tiny filaments form unregular networks of entangled threads (Figures 9A–D). The larger and thicker filaments are found well-spread in the cave sample, where they appear partially embedded or covered by the mineral matrix (Figures 9A,B). The more prominent filaments vary in size and width and can be easily seen with the naked eye (Figure 9A). The sinuous and curved filaments form intricated networks inside the mineral substrate (Figure 9B). Some filaments have cylindrical to conical morphologies ending in an apical area attached to the mineral surface (Figures 9D,E). Such filaments have a distinctive surface pattern, showing longitudinal and subparallel or reticular texture (Figures 9D,E). While the filaments display diverse morphologies, their chemical composition is relatively invariable, showing a high content in C and Si (Figure 9E). The delicate and tiny filaments (Figures 10A–D) occur as 10 micron-sized dense clusters of entangled threads, which spread irregularly over the mineral substrate or different elements of microbial origin. The EDS microanalysis shows that the thread networks are mainly composed of C and Si in the same way as the large filaments.

**Figure 11:**
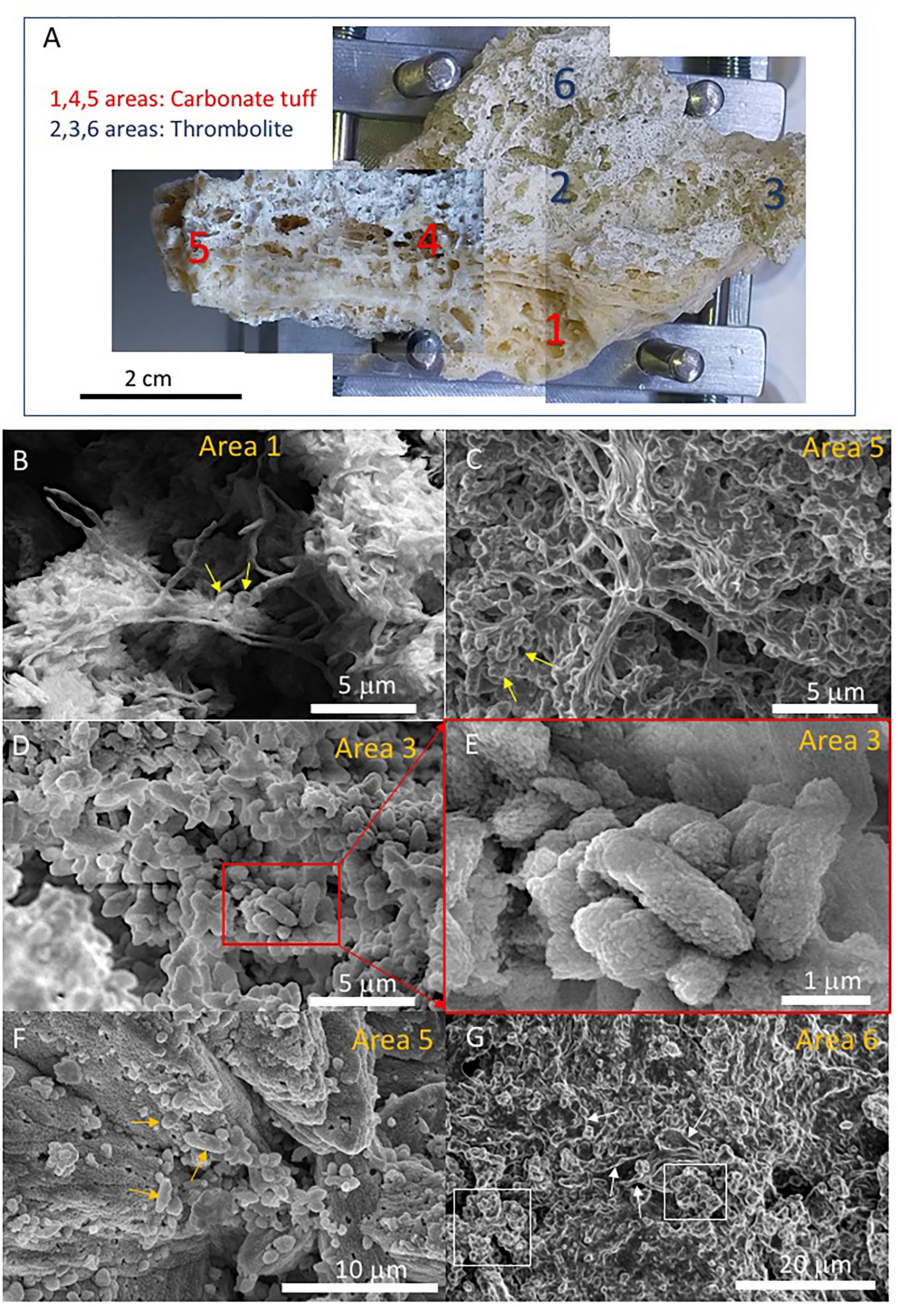
Microscope images of samples 134–3 obtained using the SEM Philips XL30 powered with a Field Emission Gun scanning large samples up to 8 cm. **(A)** Mosaic of pictures captured from the SEM visible camera showing the different scanned areas. The sample reveals two main different sedimentary fabrics, including a tuff-like carbonatic material with large filamentous elements (scanned areas 1, 4, and 5) and microbially built deposits (scanned areas 2, 3, and 6). SEM images in areas 1 and 5 **(B,C)** likely displaying mineralized branching hyphae bearing sporangia. **(D)** Scanning image in area 3 revealing 2-micron size rod-like microstructures^©^ corresponding with mineralized bacteria. **(F)** Large filaments associated with rod-like bacteria (yellow arrows) mineralized by Mg-calcite. **(E)** Image on area 6 showing clumps of rod-like microstructures (white squares) associated with void filamentous elements (white arrows) suggesting mineralization of cell wall followed by organic degradation. However, the captures a low intensity from the secondary electron signal that agrees with a composition dominated by carbon. **(G)** The thrombolite fabric follows another internal arrangement in form of clumps of rod-like microstructures associated with void filaments.

The SEM analysis for sample 134–3, associated with the carbonate outcrops of the Chiquini cave entrance, exposed a quite different record of microstructures. As described before, 134–3 show two distinctive fabrics: a tuff-like and a thrombolitic structure (Figures 11A–G, Supplementary Figures 2A–C). The SEM imaging of the tuff level unveils the abundance of mineralized filaments (Figures 11A–C) with a branching growth, while the thrombolytic area shows a high population of micron-sized and rod-like microstructures (Figures 11D,E). Interestingly, the rod-like microelements are also found on the surface of >10-micron thick mineralized filaments (Figure 11F) in the 134–3 tuff fabric. The thrombolite fabric follows another internal arrangement in form of clumps of rod-like microstructures associated with void filaments (Figure 11G).

### Lipid Analysis

**Figure 12:**
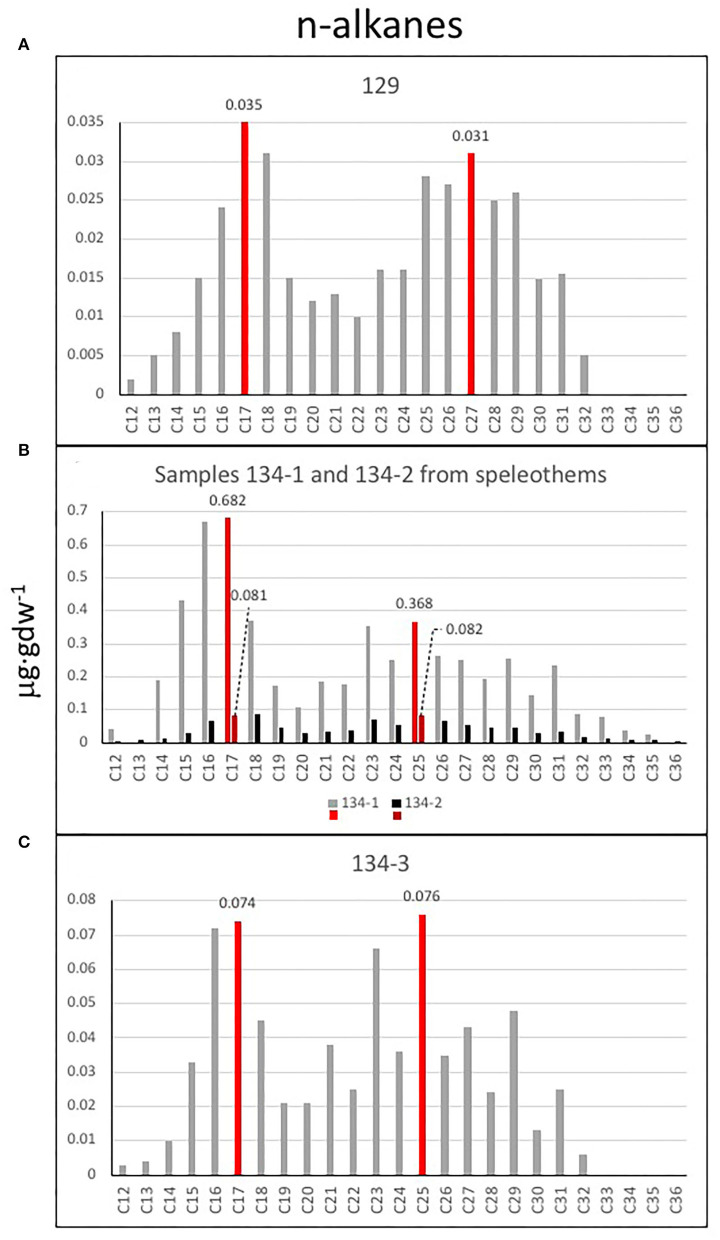
Bar diagrams plotting the C_12_-C_36_ *n-*alkane distribution in samples **(A)** 129, **(B)** 134–1 and 134–2, and **(C)** 134–3. In general, the diagram profiles reveal two different sources for *n*-alkanes. A dominance of microbial remnants (<C_20_) with the major peaks indicating potential relevance of cyanobacteria (C_15_ and mostly C_17_) among other microorganisms represented by *n*-alkanes C_16_ and C_18_; and a second group of relative high peaks in C_23_ and C_25_ suggest a source in macrophytes and/or mosses, as well as higher plants (C_27_, C_29_, and C_31_). Sample 134–1 **(B)** has the largest concentration of *n*-alkanes suggesting a higher microbial activity.

**Figure 13:**
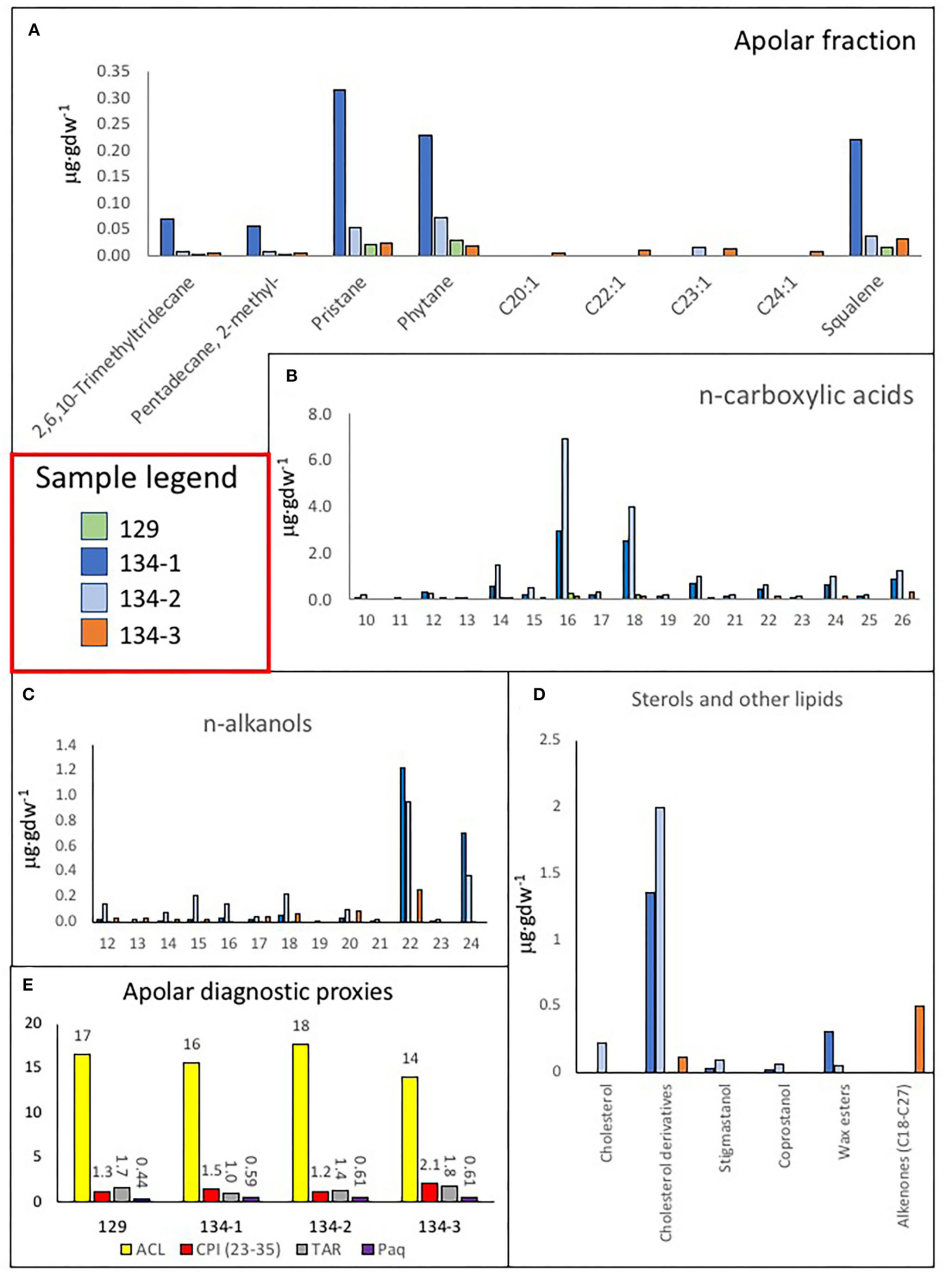
Bar diagrams revealing the occurrence and distribution of different lipids. **(A)** Concentration of some lipids extracted in the apolar fraction like trimethyl-tridecane, methyl-pentadecane, pristane, phytane, squalene, eicosene (C_20 : 1_), docosene (C_22 : 1_), tricosene (C_23 : 1_), and tetracosene (C_24 : 1_). **(B)** Distribution of *n*-fatty acids from C_12 : 0_ to C_26 : 0_ revealing a similar distribution in all samples suggesting the predominance of microbial sources in all samples (<C_20 : 0_, mostly C_16 : 0_ and C_18 : 0_) but 134–3, where the eukaryotic source (C_26 : 0_) seems to be relatively higher; interestingly, sample 129 has only recorded microbial sources (C_14 : 0_, C_16 : 0_, and C_18 : 0_). **(C)** Distribution of C_12_-C_24_ alkanols in the four samples, where a dominance of mid-molecular weight alkanols (C_22_-C_24_) was found in all samples denoting a eukaryotic source like fungal, algal, or macrophyta, but sample 129 with a stronger microbial signal. **(D)** Bar diagram displaying the concentration of cholesterol, cholesterol derivatives (cholestenone and cholestanol), wax esters (lauryl stearate, myristyl stearate and cetyl stearate) (see Supplementary Table 2) and C_18_-C_27_ alkenones in the four samples 129, 134–1, 134–2, and 134–3. **(E)** Apolar compound ratios like the average chain length (ACL), the carbon preference index (CPI), the terrigenous over aquatic ratio of hydrocarbons (TAR), and the P_aq_ proxy for submerged/floating aquatic macrophyte input vs. emergent and terrestrial plant estimated reveal the organic source in samples.

The analysis of the three lipidic fractions (non-polar, acidic, and polar) extracted from the samples collected in the cave and lacustrine deposits revealed the presence of diverse lipid families, including *normal* (i.e., straight and saturated), branched (i.e., with methyl groups), and unsaturated (i.e., with double bonds) chains (Supplementary Table 2). The non-polar fraction was mostly composed of *n*-alkanes from 12 to 36 carbons that showed a molecular distribution with a general maximum at C_17_ except for the sample 134-2 (max. at C_18_), and secondary peaks at C_25_ (samples 134–1, 134–2, and 134–3) or C_27_ (sample 129) (see Figure 12). Sample 134–1, which is a finely laminated fragment from a speleothem, showed the largest concentration of *n*-alkanes and a similar molecular profile to that of sample 134–3 (Figures 12B,C). Other compounds detected in the non-polar fraction were the isoprenoids pristane, phytane, and squalene, which were particularly abundant in sample 134–1 (Figure 13A, Supplementary Table 2).

The acidic fraction contained *n*-fatty acids ranging from 10 to 26 carbons (Figure 13B) in concentrations 1–2 orders of magnitude higher than the *n*-alkanes (Supplementary Figure 4A). They were particularly abundant in samples 134–1 and 134–2, where *n*-C_16 : 0_ and *n*-C_18 : 0_ were prevailing among the generally predominant even short chains (i.e., >20 carbons). In sample 129, only the *n*-fatty acids C_14 : 0_, C_16 : 0_, and C_18 : 0_ were detected. Other compounds found in the acidic fraction were monounsaturated fatty acids of 16 and 18 carbons [C_16:1(ω7)_, C_18:1(ω5)_, C_18:1(ω8)_, and C_18:1(ω9)_], branched fatty acids of *iso*/*anteiso* (*i*/*a*) configurations (methyl groups at ultimate or penultimate positions, respectively) from 12 to 17 carbons (*a*C_12_, *i*C_14_, *i*/*a*C_15_, *i*C_16_, and *i*C_17_), the 10-methyl hexadecanoic acid (10Me–C_16 : 0_), and a few ketones (Figure 13B, Supplementary Figures 4B,C, Supplementary Table 2). Interestingly, only *n*-fatty acids were found in sample 129, whereas the branched fatty acids were only present in samples 134–1 and 134–2 (Supplementary Figure 4B).

The polar fraction was primarily composed of *n*-alkanols (Figure 13C) with chains from 12 to 24 carbons of even-over-odd preference. In contrast to the *n*-alkanes and *n*-fatty acids, the *n*-alkanols series show maximum peaks at compounds of larger chains (C_22_ and C_24_) in all samples but 129, whose content of *n*-alkanol was generally low. Other compounds found in the polar fraction were sterols like stigmastanol, coprostanol, and cholesterol, and various derivatives (cholestenone or cholestanol) were also found in the polar fraction (Figure 13D, Supplementary Table 2). Most sterols occurred in samples 134–1 and 134–2, or those with fragments of finely laminated structure (Figure 13D, Supplementary Figures 1B,C, Supplementary Table 1). Finally, a series of alkenones from C_18_ to C_27_ were also found only in sample 134–3, the carbonatic tuff (Figure 13D).

Several lipid ratios were calculated (Figure 13E, Supplementary Figure 4D) to trace back biosources and environmental conditions. The average chain length (ACL) of *n*-alkanes informs about the dominance of prokaryotic (≤20) or eukaryotic (>20) sources (van Dongen et al., 2008), and here, it was found to range from 14 (134–3) to 18 (134–2) (Figure 13E). The carbon preference index (CPI) of the *n*-alkane is a proxy for the extent of biomass degradation, where living plants commonly have values of >5 (Rielley et al., 1991) and approaching 1 with increasing maturity. In the Uyuni samples, all values were higher than 1, especially in samples 134–3 (Figure 13E), which denotes an odd-over-even predominance of long-chain terrestrial compounds (Hedges and Prahl, 1993). The P_aq_ ratio traces the input of vegetal material from submerged/floating aquatic macrophytes vs. those of emergent and land plants (Ficken et al., 2000), and here, it was found to be between 0.44 (sample 129) and 0.61 (134–2 and 134–3). The terrigenous-over-aquatic ratio [defined as TAR = (C_27_ + C_29_ +C_31_)/(C_17_+ C_19_ + C_21_); Bourbonniere and Meyers, 1996] was higher than one in all samples but 134–1 (Figure 13E). The ratio of pristane over phytane (Pr/Ph), two compounds largely derived from chlorophyll-a (Didyk et al., 1978), is commonly used to discriminate between oxic (>1) or anoxic (<1) conditions in a deposition environment (Peters et al., 2005), and here, it was observed to be higher than one in samples 134–1 and 134–3, and lower than one in samples 129 and 134–2 (Supplementary Figure 4D).

## Discussion

**Figure 14:**
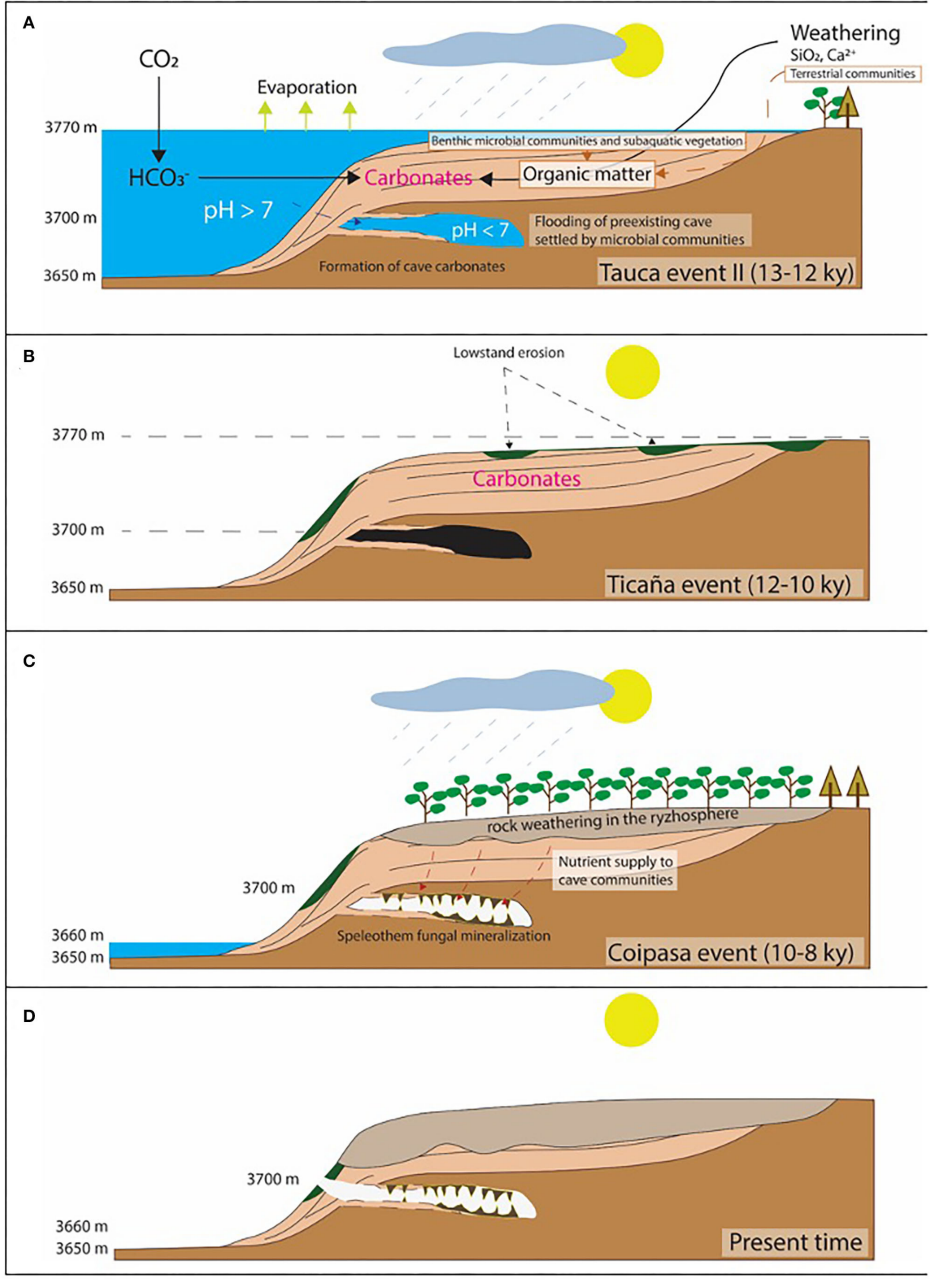
Sketch describing the stages leading to the formation of the carbonate structures in the cave interior consisting of thrombolite-like and speleothems framed by fungal colonies. **(A)** Flooding of a cave initially emplaced in the volcanic deposits around Tunupa volcano. It was caused by a highstand episode reaching an altitude of 3,770 m corresponding with the wet Tauca event (13–13 ky), promoting the precipitation of lacustrine and cave carbonates with thrombolitic fabric mediated by microbes. **(B)** The lake level dramatically dropped during an aridic incursion (Ticaña event) associated with an erosive fluvial activity. **(C)** Subsequent wet episode (Coipasa event) leading to a highstand stage lower than the cave bottom greatly enhanced the weathering of the lacustrine carbonates and other exhumated deposits. The hydrological activity triggered the forest expansion in the lake margins, augmented substrate weathering, incremented the ecosystem productivity and the nutrient circulation from the surface to the cave interior. Such processes sustained the fungal community in the cave, which activity ended in the speleothem formation. **(D)** A last aridic event in the Holocene collapsed the Uyuni hydrological activity that formed the modern saline basin. Under these conditions, the cave community was obliterated, and the speleothem formation ended.

The structure and distribution of the speleothems that are framed with large filamentous structures are consistent with their formation during a wet and alteration episode that affected the lacustrine carbonates topping the volcanic deposits. As observed *in-situ* (Figures 2B,E), the cave ceilings are composed of volcanic deposits that are covered by a first carbonate unit showing a short-branched fabric found in the lacustrine thrombolitic carbonates of Pleistocene (Figures 2B,E, Supplementary Figures; Rouchy et al., 1996; Placzek et al., 2006). As it has been observed, such unit is followed by other two with distinctive filamentous structures that correspond to dense hyphae networks that have been mineralized by Mg-calcite as identified through XRD (Supplementary Figure 3B). The sequence of those carbonate units agrees with: (1) the flooding of a pre-existent cave emplaced in the volcanic host rock by a highstand lacustrine episode, followed by (2) a low-standing pulse, which finally (3) ended with the speleothem formation by a novel wet lacustrine episode not high enough for flooding the cave (Figures 14A–D). It resulted from the evolution of the Salar de Uyuni during the late Pliocene to the early Holocene. First, the saline basin experienced a highstand (>3,700 mls) episode in the Tauca event (>12 ky). Then, it was followed by a dramatic drop in the lake level during an arid phase in the Ticaña event (>10 ky) and ended by the Coipasa wet episode (10–8 ky). Interestingly, the cave has recorded carbonatic materials that formed under quite different paleoenvironmental conditions. During the Tauca highstand, the cave flooding sustained a cryptic habitat where likely the sunlight was a limiting factor for the photosynthetic microbial communities that might change to heterotrophic and/or chemosynthetic in the deeper areas of the caves. The environmental conditions changed drastically when the Coipasa episode took place, which favored the emplacement of a karst system through an active hydrological cycle.

The sedimentary context, fabric, and structure of the different samples are consistent with the paleoenvironmental conditions where they were formed (Supplementary Figures 1, 2, Supplementary Table 1). The columnar fabric found in sample 129 collected from lacustrine carbonates of the Coipasa area (Supplementary Figure 1A, Supplementary Table 1) suggests that is the result of the microbial clot accretion resulting in the generation of a columnar thrombolite fabric in a lake with brackish to saline waters (Rouchy et al., 1996; Placzek et al., 2006). In turn, the laminar and leafy internal structures observed in samples 134–1, 134–2, and 134–3 concur with the precipitation of Mg- calcite (Supplementary Figures 3B,C, Supplementary Table 1) by different microbial and non-biological pathways, where fungi played an essential role in the ion mobilization and the speleothem formation. The XRD analysis suggests that the mineral precipitated occurred under varying saline solutions (Risacher and Fritz, 1991) leading to the production of Mg-calcite. Furthermore, the occurrence of kutnahorite Ca_1.1_(Mn^2+^, Fe^2+^, Mg)_0.9_(CO_3_)_2_ in samples 134–3 (Supplementary Figure 3C) concur with the carbonate precipitation under microbial reduction of iron and manganese in the ancient lacustrine and ground solutions enriched with carbonate and magnesium (Rincón-Tomás et al., 2016).

The SEM-EDAX analysis of the different samples has provided additional information about the forming paleoenvironmental conditions through their geochemical and biological composition. The SEM-EDS analysis of samples 134–1, 134–2, and 134–4 corresponding to the speleothems show that the mineral matrix has a fibrous radial habit (Figures 3, 4A), which is composed of Ca, C and O, and secondary Mg (Figure 6A). It greatly agrees with the occurrence of the Mg-calcite composition identified by XRD. Furthermore, the mineral matrix comes together with other elements like Fe, Cu, Mn, and Si (Figures 6C,D) suggesting that they are sourced from the degradation of the volcanic host rock that is the Uyuni basin basement (Tibaldi et al., 2009; Salisbury et al., 2015). Such a set of elements are abundant in very distinctive microstructures like the spongy ovoids and undulate laminas bearing Mn, and the aggregates of rod-like units that are mineralized by Si (Figures 4B–D, 5A,B). The Mn-enriched microstructures suggest that kutnahorite and Mn oxides locally formed through microbial reduction of Mn and Fe in the speleothems (Rincón-Tomás et al., 2016), while the silica-rich aggregates of rod-like elements can result from the silicification of bacteria biofilm (Toporski et al., 2002; Moore et al., 2020) by circulating solutions within the speleothem.

The SEM-EDS technique has also identified small and delicate tests, with an orderly distribution pattern and geometry (Figures 7A–F). The tests have a flask-shaped form and on one of the sides, there is an opening or hexagonal apertural margin surrounded by indentations evenly distributed around the aperture and containing several teeth (Figure 7A). The SEM-EDS analysis shows that they are built by the coalescence of different units composed of silica (Figures 7C,D). Such a microstructure test fits well with the testate amoeba carapace, which is formed by platy units known as idiosomes with silica composition (Lahr et al., 2015). Amoeba is unicellular organisms that normally live protected by a test in both subaerial or subterranean environments (González López et al., 2013). In the subterranean environment, the tests are normally formed by amorphous silica and agglutinated idiosomes that resemble pollen grains contained inside the organism (González López et al., 2013).

The speleothems, formed by the precipitation of carbonates coming from the rock dissolution due to chemical weathering, should have created a suitable and organic-rich environment for the testate amoebae to thrive. The testate amoebae were found on the external surface of speleothems protected inside small depressions or inside speleothem micropores (Figure 7G). The tests appear mostly intact, although a few specimens were found with the tests partially collapsed by flattening or dismantled where the idiosomes are released (Figures 7A,C,D). The specimens appear as isolated individuals and the great homogeneity in the distribution of the testate amoebae indicates that the microsystem inside the cave was very similar for the time of their formation, as any changes in the humidity levels, availability of silica, or any transport process would undoubtedly affect the formation of the test and inhibiting the occurrence of such protozoans. There is extensive literature describing amoebae; however, they are normally referred to as naked amoebae (without tests). The observations made in several samples allowed us to elaborate on the origin of the testate amoebae in the Uyuni caves, however, it is obvious that more samples should be studied, both in the origin zones and in the number of speleothems.

Furthermore, the siliceous thecae co-occurring with the testate amoeba have the same morphological and compositional features as diatom frustules (Figures 8A–D). They have also been found in the ancient deposits of the Uyuni basin (Servant-Vildary, 1978). The appearance of such remains in the speleothem samples (134–1, 134–2, and 134–4) suggests that diatoms were a biological component of the Uyuni cave ecosystems. Interestingly, cave diatoms have been found associated with different karstic structures, including the speleothems (Kashima et al., 1987; Falasco et al., 2014), in the same way as they have in the speleothems of the Uyuni caves (Figures 6A–D). The distribution of the diatoms in the cave should correspond to the cave topology, which is the main constraint for the light availability in the cave interior (Falasco et al., 2014). The diatom frustules show different morphology and preservation degrees depending on the site where they are found. While the specimens occurring in the external sheet of the speleothem samples (e.g., 134–4) are intact and have an asymmetric morphology (Figures 8A,B), those observed in the internal lamina are symmetric and fragmented, have traces of dissolution, and are partially filled and covered by carbonatic material (Figures 8C,D, Supplementary Figure 5). Interestingly, the morphology and preservation degree of the diatom frustules in sample 134–4 from the carbonate tuff (Supplementary Figure 5, Supplementary Table 1) shows the same features (e.g., internal mineralization, dissolution traces) as those found in the internal lamina of the samples collected from the speleothems. Consequently, the microbial remains, including the diatoms that are found on the surface of the external mineral sheet (Figures 3A–D, 4A, 8A,B), are relatively younger than the mineral matrix that contains them.

The observation through the SEM of straight to slightly sinuous long (>1 mm) filamentous structures of samples 134–4 (Figures 9A,B) shows that they are partially or fully mineralized by Mg-calcite, suggesting that they have grown before or when the mineral precipitated. In this regard, the macrofilaments forming the speleothems show that their features are widely recognized in typical fungi hyphae as thick filaments and a high branching degree (Figures 2B–E, Supplementary Figure 2). As these are completely mineralized by the Mg-calcite, it can be inferred that the cave fungal community played an essential role in the speleothem formation. On the contrary, the entangling threads forming unregular networks (Figures 10A–D) resemble the extracellular polymeric substances (EPS) of microbial biofilms (Dohnalkova et al., 2011). The size and morphology of the EPS threads are consistent with a bacterial source as it is shown by Dohnalkova et al. (2011) and references therein.

Furthermore, the preservation degree and mineralization of filaments and EPS are also variable. In sample 134–1, they show a high content of organic carbon and incipient mineralization by silica (Figures 9I, 10C,D). In turn, sample 134–3 show high mineralization by carbonate (Figures 11A–E) and, likely, silica (Figure 11G) with a varying concentration in C. Such disparate preservation can be a consequence of the different sample and microstructure ages; while in samples 134–1 and 134–4, the external sheet contains the elements with a higher preservation degree (Figures 8A,B), which have lower preservation and high mineralization in the internal lamina (Figures 8C,D, Supplementary Figure 5). This is also the case for the microbial remains in sample 134–3, where the diatoms appear fragmented and with clear evidence of corrosion (Supplementary Figure 6).

The molecular distribution of the *n*-alkane series revealed that the dominance of microbial remnants (<C_20_) is likely related to cyanobacteria (peaks at C_15_ and mostly C_17_) (Ladygina et al., 2006), among other microorganisms [ubiquitous *n*-C_16_ and *n*-C_18_]; (Grimalt and Albaigés, 1987; Figures 12A–C). The prevailing microbial sources were reflected by homogeneously low values of ACL in the four samples (14–18) (Figure 13E, Supplementary Figure 4D). In particular, the signal of cyanobacteria (as *n*-C_17_) was prevailing in all samples but 134–2, where other microorganisms (as *n*-C_18_) appeared relatively more abundant (Figure 12B). Furthermore, the widespread presence of 2-methylpentadecane (Figure 13A) supported the relevant contribution of cyanobacteria in the samples (Brocks and Summons, 2014). Still, there was also a presence of eukaryotic signals related to diatoms and algae [possibly C_16:1(ω7)_ and C_18:1(ω9)_ fatty acids], mosses, and macrophytes (*n*-C_23_ and *n*-C_25_ alkanes; Ficken et al., 2000; Pancost et al., 2002), and higher plants (*n*-C_27_, *n*-C_29_, and *n*-C_31_ alkanes; Eglinton and Hamilton, 1967; Hedges and Prahl, 1993). As a result, three of the four samples (129, 134–2, and 134–3) showed TAR values slightly higher than 1 (Figure 13D), which revealed that the proportion of aqueous biomass (algae and cyanobacteria), as represented by the *n*-alkanes C_17_, C_19_, and C_21_, was lower than that derived from higher plants (as the sum of C_27_, C_29_, and C_31_), except for the sample 134–1 (i.e., TAR = 0.8).

Still, sample 134–1 showed together with the rest of the samples' CPI values higher than one that denoted an odd-over-even predominant character, slightly higher in 134–3 (Figure 13E, Supplementary Figure 4D). This may be interpreted in relation to the still fresh nature of the terrigenous long-chain *n*-alkanes, likely due to good preservation of the vegetal biomass after death (Carrizo et al., 2019). This was supported by the relative enrichment of *n*-fatty relative to *n*-alkanes observed in all samples (Supplementary Figure 4A). The particularly high ratio of n-fatty/n-alkanes in 134–2 (ratio of 19) implies a much lower defunctionalization of the organic matter over time (i.e., loss of functional groups) relative to the other samples. Furthermore, it cannot be discarded that some organics could be very recent as resulting from later microbial activity in the cave as observed through the SEM-EDS in sample 134–1 (Figures 8A,B, 9A–I). This is consistent with a younger age for sample 134–2 collected from the fungal-framed speleothems.

Regarding aqueous sources, values of the P_aq_ index from 0.44 (sample 129) to 0.61 (samples 134–2 and 134–3) suggested organic matter inputs from a mix of emergent and submerged/floating macrophytes (Ficken et al., 2000), as well as mosses (Nott et al., 2000). The presence in the apolar fraction of other compounds like the isoprenoids pristane, phytane, and squalene (Figure 13A) was related mostly to photosynthetic sources. Pristane and phytane are largely derived from the degradation of phytol, a side chain of chlorophyll-a mostly used by phototropic organisms like cyanobacteria, algae, and land plants (Rontani and Volkman, 2003; Peters et al., 2005), while squalene is practically ubiquitous in all type of organisms, including animals (Grice et al., 1998; Brocks and Summons, 2014). Assuming a common origin in phytol of both pristane and phytane, we can learn about the deposition environment of the samples by calculating the ratio of one over the other (i.e., Pr/Ph; Peters et al., 2005). Here, values of the ratio from 0.7 to 1.4 (Supplementary Figure 4D) allowed us to differentiate anoxic environment for samples 134–1 and 134–3 (ratio >1) and an anoxic environment for samples 129 and 134–2 (ratio > 0.7) (Powell and Mckirdy, 1973). Changes in paleoenvironmental conditions may have driven periodic transitions from dry to lacustrine systems in the cave that explain the mix of depositional environments and aqueous/terrigenous fingerprints found in the samples. In general, anoxic conditions recorded in sample 129 are consistent with the formation of lacustrine thrombolites, where anaerobic microbial communities take part in the formation of the carbonatic biostructures (Feldmann and Mckenzie, 1998). In the same way, anoxic to oxic conditions, shown in samples 134–1 and 134–2, will likely result from changing environmental conditions in the speleothem formation, while aerobic conditions are consistent with the paleoenvironment where the carbonatic tuff (sample 124–3) was formed.

In the four samples, the similar dominance of short-chain *n*-fatty acids (< C_20 : 0_, mostly C_16 : 0_ and C_18 : 0_) supported the mentioned dominance of microbial sources in the caves (Figure 13B). In particular, the detection of branched fatty acids, such as 10Me-C_16 : 0_, *a-*C_12 : 0_, *i-*C_14 : 0_, *i*/*a-*C_15 : 0_, *i-*C_16 : 0_, and *i-*C_17 : 0_, was related to *Actinomycetes* (*Actinobacteria* phylum), *Desulfobacter* (*Proteobacteria* phylum), and other sulfate-reducing bacteria (Taylor and Parkes, 1983; Parkes et al., 1993). Still, a certain eukaryotic signal was observed in sample 134–3 in form of a peak at C_26 : 0_, most likely related to algae, mosses, or aquatic macrophytes (Ficken et al., 2000; Nott et al., 2000), according to its P_aq_ value (0.61; Supplementary Figure 4D). In contrast, in samples 134–1 and 134–2, the eukaryotic signal in the acidic fraction was, instead, related to higher plants, according to the presence of oxodehydroabietic acid, a typical resin acid derived from coniferous plants (Rybicki et al., 2016; Marynowski et al., 2020). Furthermore, the presence of unsaturated fatty acids, such as C_16:1[ω7]_ and C_18:1[ω9]_ in samples 134–1, 134–2, and 134–3, might also indicate contributions from eukaryotic sources, such as aquatic diatoms, microalgae, and fungi, as well as cyanobacteria or other gram-negative bacteria (Ahlgren et al., 1992; Dijkman and Kromkamp, 2006; Coates et al., 2014), or type II methanotrophs [C_18:1(ω8)_] (Bowman et al., 1991; Brocks and Summons, 2014). The relatively higher concentration of unsaturated fatty acids in samples 134–1 and 134–2 (Supplementary Figures 4B,C, Supplementary Table 2) suggested a fresher nature of these samples agreeing with their highest *n*-fatty acids/*n*-alkanes values (Supplementary Figure 4A) since diagenesis and alteration over time tend to cause the loss of double bonds (Stefanova and Disnar, 2000).

In the polar fraction, the dominance of C_22_ and C_24_ among the *n*-alkanols series in samples 134–1, 134–2, and 134–3 (Figure 13C) supported the presence of biomass from eukaryotic sources. In particular, the relationship of *n-*C_24_ with higher plants is well-described (Peters et al., 2005; Burdige, 2007), and would agree with a potential source here in the forest communities associated with the soil formation above the speleothem. In contrast, the origin of *n-*C_22_ remains unknown, and we hypothesize that it could stem from fungi biomass. In sample 129, only compounds of microbial sources (*n*-C_14_, *n*-C_16_, and *n*-C_18_) were found in the polar fraction (Figure 13B), which agrees with a composition related to thrombolite formations by cyanobacteria (Rouchy et al., 1996).

The recovery of several sterols, including stigmastanol and cholesterol derivatives (Figure 13D, Supplementary Table 2), confirmed the presence of eukaryotic biomass. Stigmastanol is a product derived from stigmasterol, a phytosterol produced by higher plants and micro-/macroalgae (Volkman, 1986, 2003). Cholesterol derivatives, such as cholestadienone and cholestadienol (Melendez et al., 2013), and coprostanol (Figure 13D, Supplementary Table 2), suggest the occurrence of organics produced by animals, protozoa, and red algae (Volkman et al., 1998). In particular, the detection of cholestadienone (cholesta-3,5-dien-7-one) and cholestadienol (3β-cholesta-4,6-dien-3-ol) are derivatives from cholesterol that denote a low extent of degradation (Melendez et al., 2013), while coprostanol has been reported as a fecal degradation product found in mammals and birds feces (Harrault et al., 2019; Gallant et al., 2021). Among the samples, cholestadienol and coprostanol mainly occurred in samples 134–1 and 134–2, where speleothem fragments were present, whereas cholestadienone was dominant in sample 134–3, the sample with tuff-like material (Supplementary Figures 2A–C, Supplementary Table 1). Finally, alkenones from C_17_ to C_26_ were also found in the polar fraction only of sample 134–3 (Figure 13D). While long-chained alkenones (C_36_-C_38_) are attributed to planktonic unicellular algae (Pagani, 2009), the origin of chains from C_17_ to C_26_ is uncertain.

## Conclusion

The analysis of the different samples in the Salar de Uyuni provides unique information about the paleoenvironmental conditions where they were formed, by diverse microbial and non-biological pathways, where the fungal communities played a crucial role in the formation of the speleothems. Likely, nutrients were primarily provided by the vegetal communities in the lake margins, however, they may have also been released from the lacustrine carbonatic unit. The combination of biological activity and hydrology most likely triggered a quick rock dissolution and mineralization, which formed the speleothem fungal structures.

The size and abundance of the preserved fungal structures suggest a constant supply of organic matter and stable hydrological activity at the time of their formation. The high porosity and large size of cavities in the paleoterraces ensured a long-lasting and continuous fluid flow and supply of materials. All the elements necessary for the mineral formation were released during the alteration of the host rocks and supplied by percolating fluids. The biological control of element cycling is also an important factor, for instance, the silica detected in the carapace of the testate amoebae, or the diatom frustules indicates the role of the organisms in the Si cycle, where weathering and transportation of Si from the older lacustrine deposits and the soils moved through the speleothems.

The analysis of the lipids recorded in the samples has also provided some insights into the paleoenvironmental conditions accompanying the formation of the biospeleothems (samples 134–1, 134–2, and 134–4), the carbonatic tuff (sample 134–3), and the thrombolitic carbonates (sample 129). The organic compounds are mostly sourced in bacteria, but also with a high input from aquatic and terrestrial plant inputs, which were lately degraded by fungi. In this regard, the signal of cyanobacteria is dominant in all samples but 134–2, where other microorganisms could be more abundant. The occurrence of sterols like stigmastanol and cholesterol derivatives prove relevant to eukaryotic activity in the past. While stigmastanol is a stigmasterol derivative produced by higher plants and micro-/macroalgae, derivatives of cholesterol like cholestadienone and cholestadienol, and coprostanol evidence the molecular record of animals, protozoa, and red algae. The characterization of cholestadienone and cholestadienol denote a low extent of degradation of the original sterols, while coprostanol, is identified as a sterol from fecal degradation by mammals and birds in caves. In this regard, cholestadienol and coprostanol are found in the speleothem samples, which is consistent with the activity of animals in the cave. However, cholestadienone is prevailing in the carbonatic tuff suggesting a red algal origin.

Searching for biomarkers in caves allows us to understand the contribution of fungal communities to biokarst not only on Earth but also in Martian cave environments. Cave microbiology can answer questions about the limits of life and allow us to recognize the geochemical signatures of life. Given that such signatures have survived geologic uplift, we should be able to detect them on other planetary surfaces, such as Mars, if they are present. In addition, due to the absence of liquid water on the surface of Mars, extant life will likely be restricted to the subsurface, making it crucial to understand the processes, which create and preserve signatures of microbial life in cave environments.

## Data Availability Statement

The original contributions presented in the study are included in the article/Supplementary Material, further inquiries can be directed to the corresponding author.

## Author Contributions

AA and DF-R wrote the manuscript with input from TH, QH, YS, RA, NR, DC, and LS. DC and LS conducted and provided the main information regarding the lipid content found in the samples. Furthermore, AA and NR prepared and analyzed the samples under the different SEM-EDS and TEM techniques. All authors contributed to the discussion and final confection of the manuscript.

## Funding

This research has been supported by the National Key Research and Development Program of China (2021YFA0716100), project PID2019-104812GH-100CTM funded by the MICINN of Spain, and project FDCT-0005-2020-A1 funded by the Fundo de Desenvolvimento das Cientifico e da Tecnologia da RAE de Macau.

## Conflict of Interest

The authors declare that the research was conducted in the absence of any commercial or financial relationships that could be construed as a potential conflict of interest.

## Publisher's Note

All claims expressed in this article are solely those of the authors and do not necessarily represent those of their affiliated organizations, or those of the publisher, the editors and the reviewers. Any product that may be evaluated in this article, or claim that may be made by its manufacturer, is not guaranteed or endorsed by the publisher.

## Supplementary Material

Supplementary Figure 1 Pictures showing the analyzed samples like **(A)** 129 with columnar fabric collected in the Coipasa area corresponding to a thrombolitic carbonate, **(B)** 134–1 and **(C)** 134–2 laminated samples recovered from a large speleothem in the Chiquini cave. Black color in sample 134–1 is found in distinct sides of the speleothems and likely corresponds with Mn-bearing minerals as detected by SEM-EDS and XRD techniques.

Supplementary Figure 2 Images displaying **(A)** sample 134–3 that has **(B)** thrombolitic and **(C)** filamentous tuff-like fabrics. **(D)** Corresponds with sample 134–4 collected from a speleothem showing a leafy fabric with internal microlaminar structure and the occurrence of filamentous networks with forked pattern (red squares) and large filaments (red arrow).

Supplementary Figure 3 XRD analysis for samples **(A)** 129, **(B)** 134–1 and 134–2, and **(C)** 134–3 resulting in different Mg-calcite species with varying content in such a cation. In addition, a Mn-bearing carbonate like kutnahorite **(C)** has been found in sample 134–3.

Supplementary Figure 4 Additional diagrams of different organic compounds revealing **(A)** a higher abundance of *n*-fatty acids over the *n*-alkanes, **(B)** the diversity of fatty acids in samples, **(C)** iso/anteiso, monounsaturated and branched fatty acids, and **(D)** the full set of apolar diagnostic ratios, including the average chain length (ACL), the carbon preference index (CPI), the terrigenous over aquatic ratio of hydrocarbons (TAR), terrestrial index from *n*-C29 over *n*-C27 alkane (C29/C17), P_aq_ proxy for submerged/floating aquatic macrophyte input vs. emergent and terrestrial plant, pristane vs. phytane (Pr/Ph), *n*-C17 alkane vs. pristane (C17/Pr), and *n*-C18 alkane vs. phytane (C18/Phy).

Supplementary Figure 5 Fragmented valve of a diatom found in the internal lamina of sample 134–4 collected from a speleothem that is infilled by carbonate sediment.

Supplementary Figure 6 Diatom frustule found in area 4 of sample 134–3 covered by mineralized filaments (yellow arrows) likely of fungal origin. The diatom valve is fragmented and reveals evidence of partial corrosion.

Supplementary Table 1 Description of the samples collected in the Salar de Uyuni for this study.

Supplementary Table 2 List of compounds found in the Uyuni samples.
