# Supplementary material for: Biospeleothems Formed by Fungal Activity During the Early Holocene in the “Salar de Uyuni”
Source: Front Microbiol. 2022 Jun 23;13:913452. doi: 10.3389/fmicb.2022.913452 (PMC9260512; doi:10.3389/fmicb.2022.913452)
Supplement: Supplementary Figure 1 — Pictures showing the analyzed samples like (A) 129 with columnar fabric collected in the Coipasa area corresponding to a thrombolitic carbonate, (B) 134–1 and (C) 134–2 laminated samples recovered from a large speleothem in the Chiquini cave. Black color in sample 134–1 is found in distinct sides of the speleothems and likely corresponds with Mn-bearing minerals as detected by SEM-EDS and XRD techniques. [file Data_Sheet_1.PDF]

Supplementary Material

Endokarstic edifices formed by fungal activity during the early Holocene in the "Salar de Uyuni"

Angélica Anglés[1,2,3], Qitao Hu[1,2], Laura Sánchez García[4], Daniel Carrizo[4], Nuria Rodriguez[4], Ting Huang[1,2], Yan Sen[1,2], Ricardo Amils[4], David C. Fernández-Remolar[1,2,5]

[1]State Key Laboratory of Lunar and Planetary Sciences, Macau University of Science and Technology, Macau 999078, SAR China

[2]CNSA Macau Center for Space Exploration and Science, Macau 999078, PR China

[3]Blue Marble Space Institute of Science, 600 1st Avenue, 1st Floor, Seattle, Washington 98104, USA

[4]Centro de Astrobiología (INTA-CSIC), Madrid, Spain

[5]Carl Sagan Center, The SETI Institute, Mountain View, CA 94043, USA

*** Correspondence:**
Angélica Anglés
angelica.angles@bmsis.org

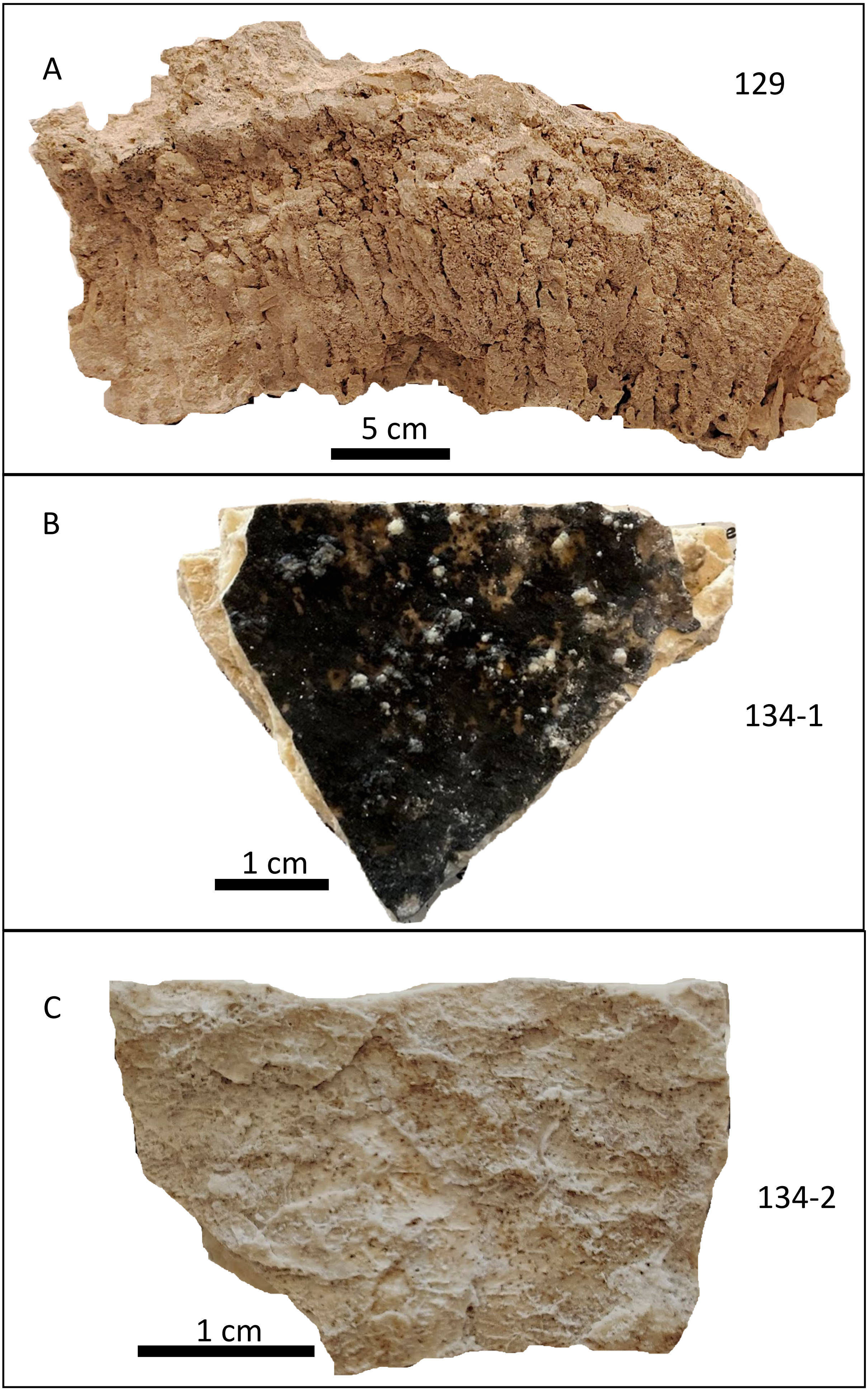

Supplementary Figure 1

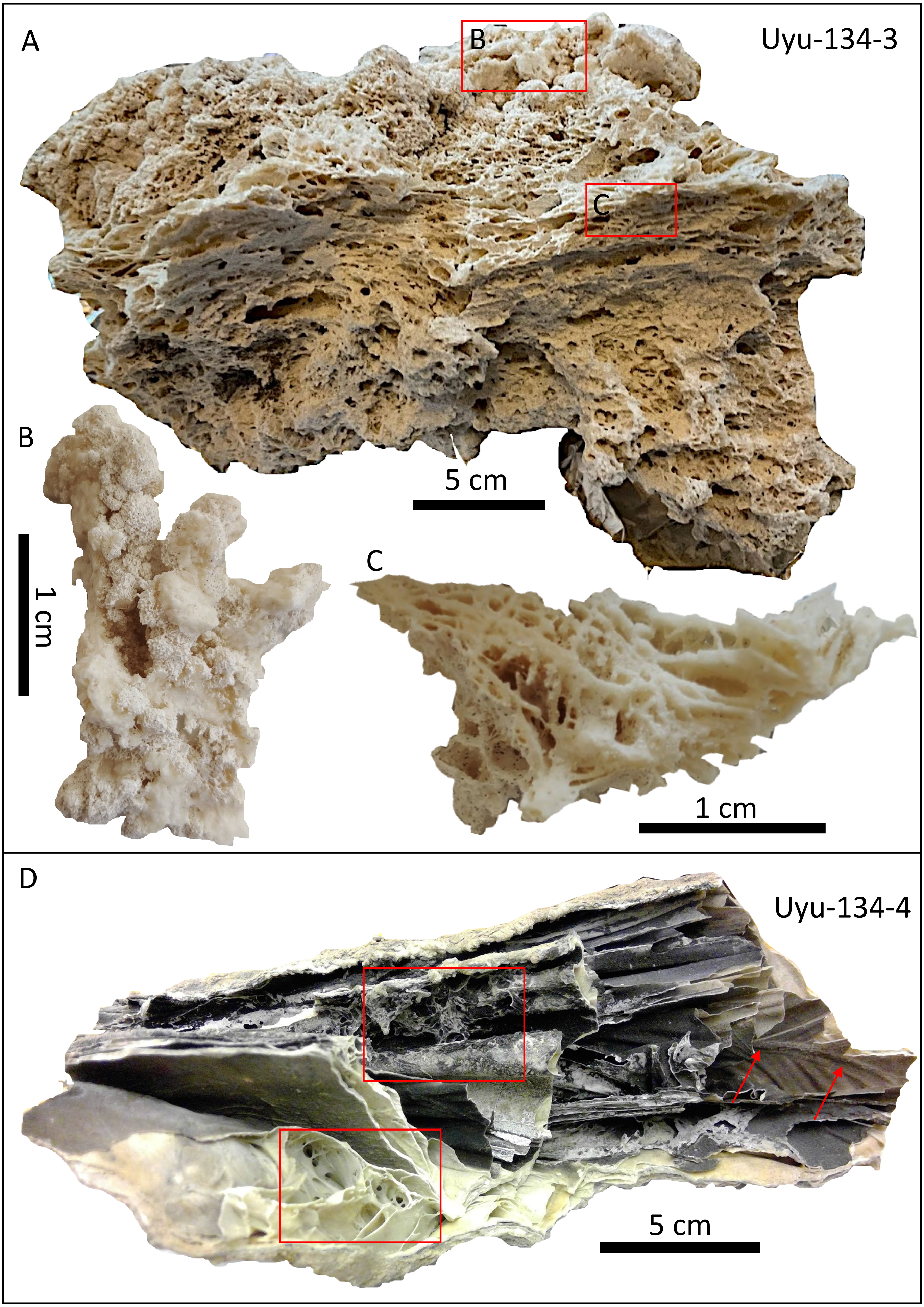

Supplementary Figure 2

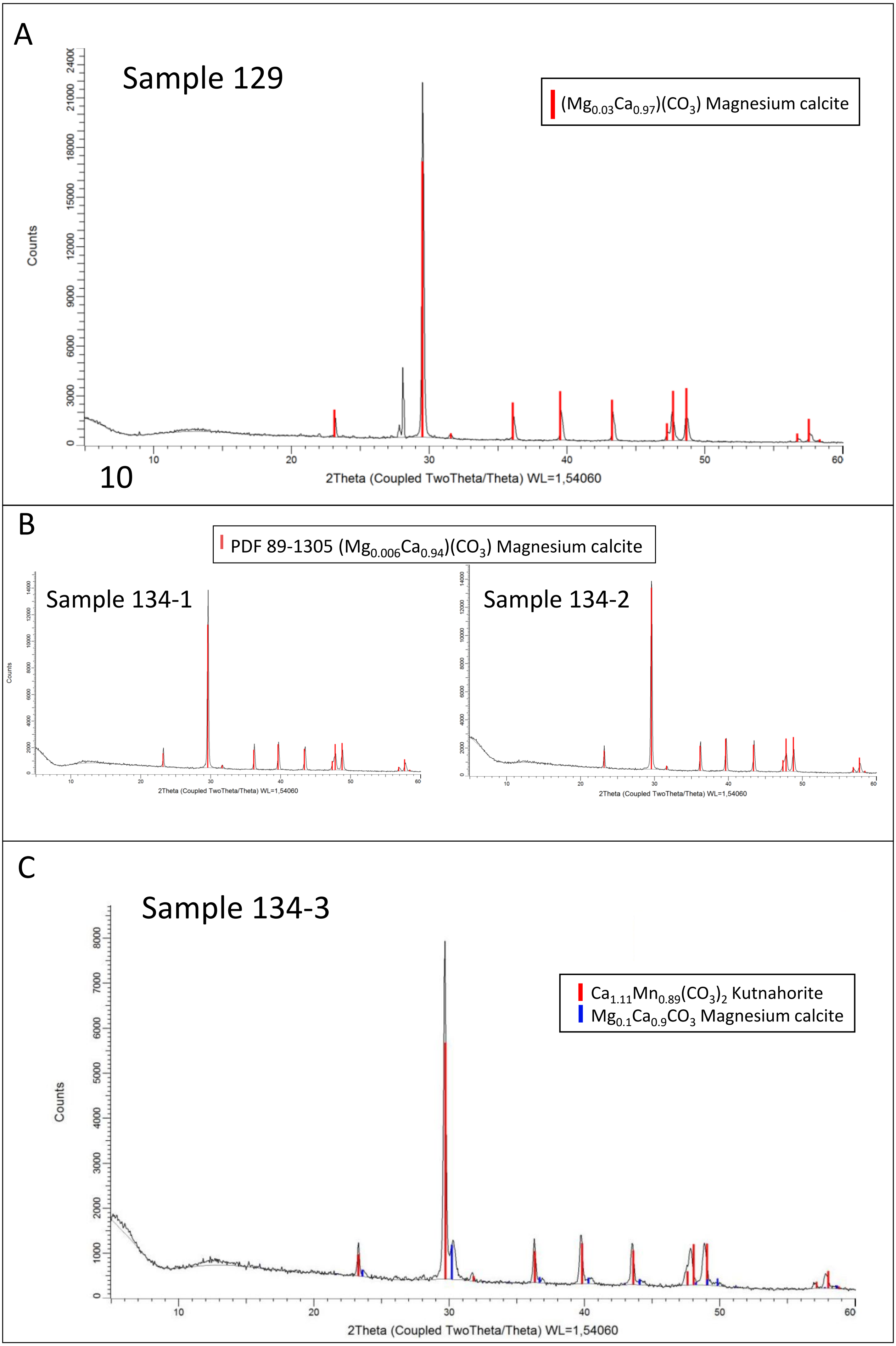

Supplementary Figure 3

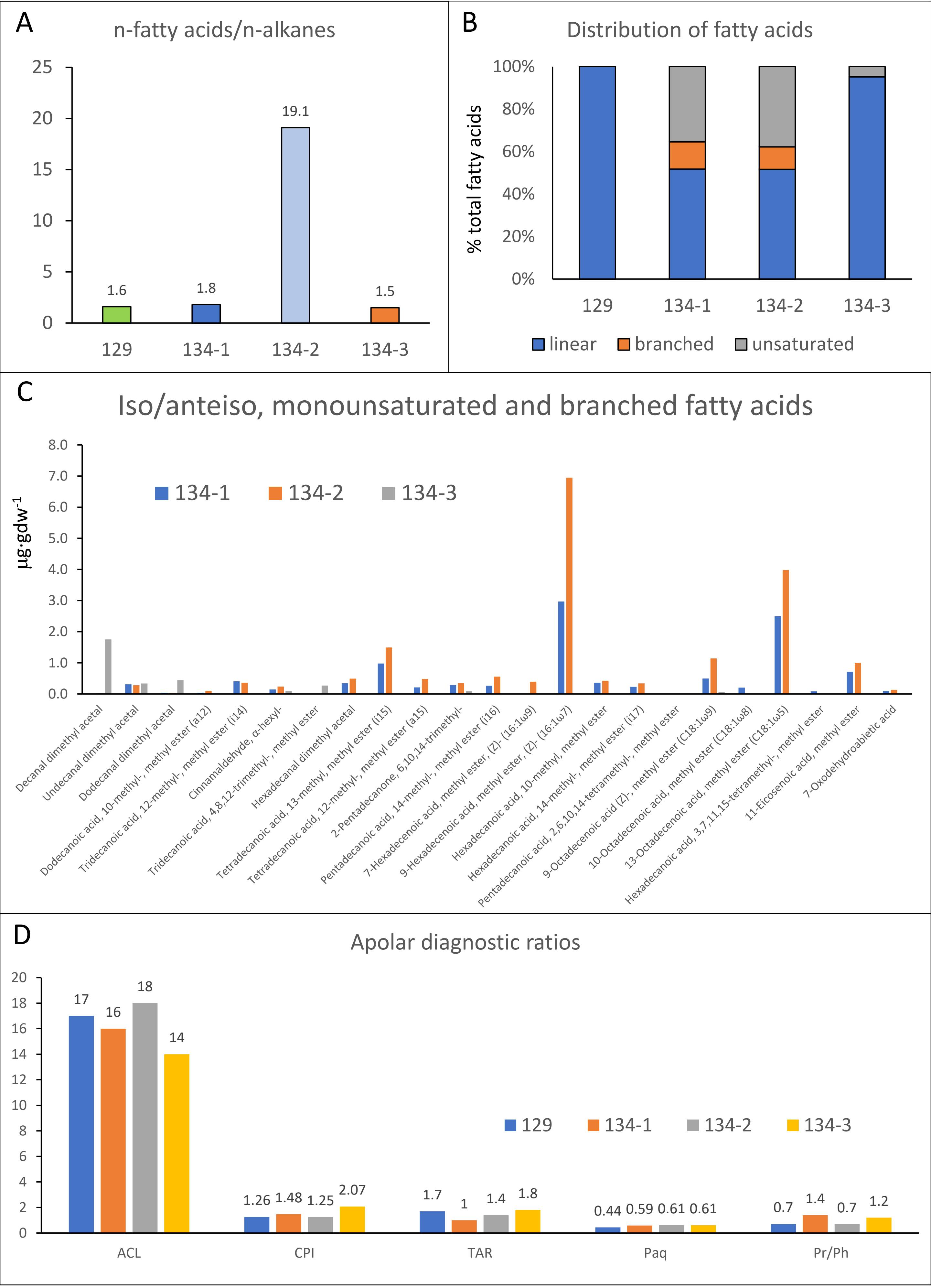

Supplementary Figure 4

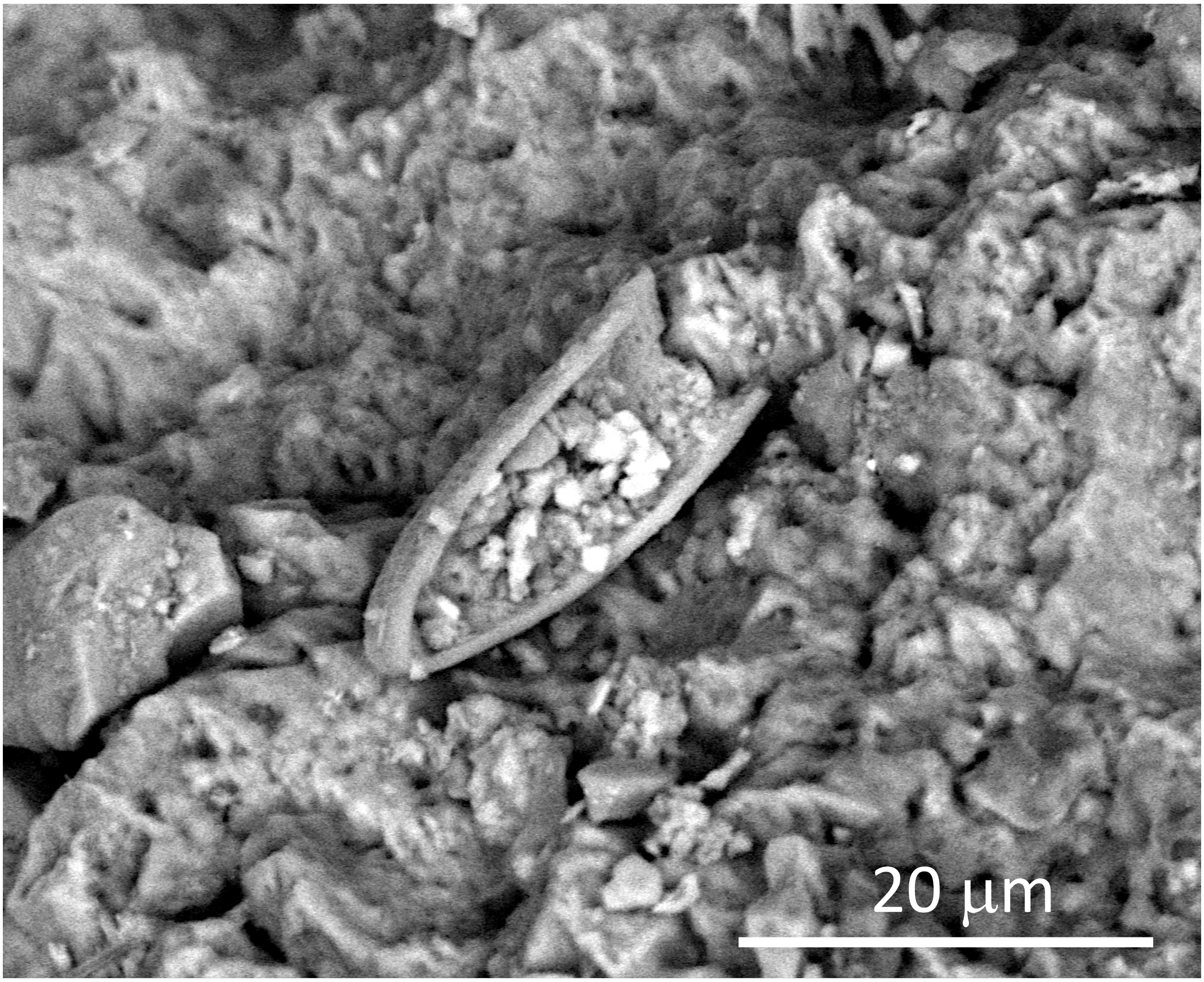

Supplementary Figure 5

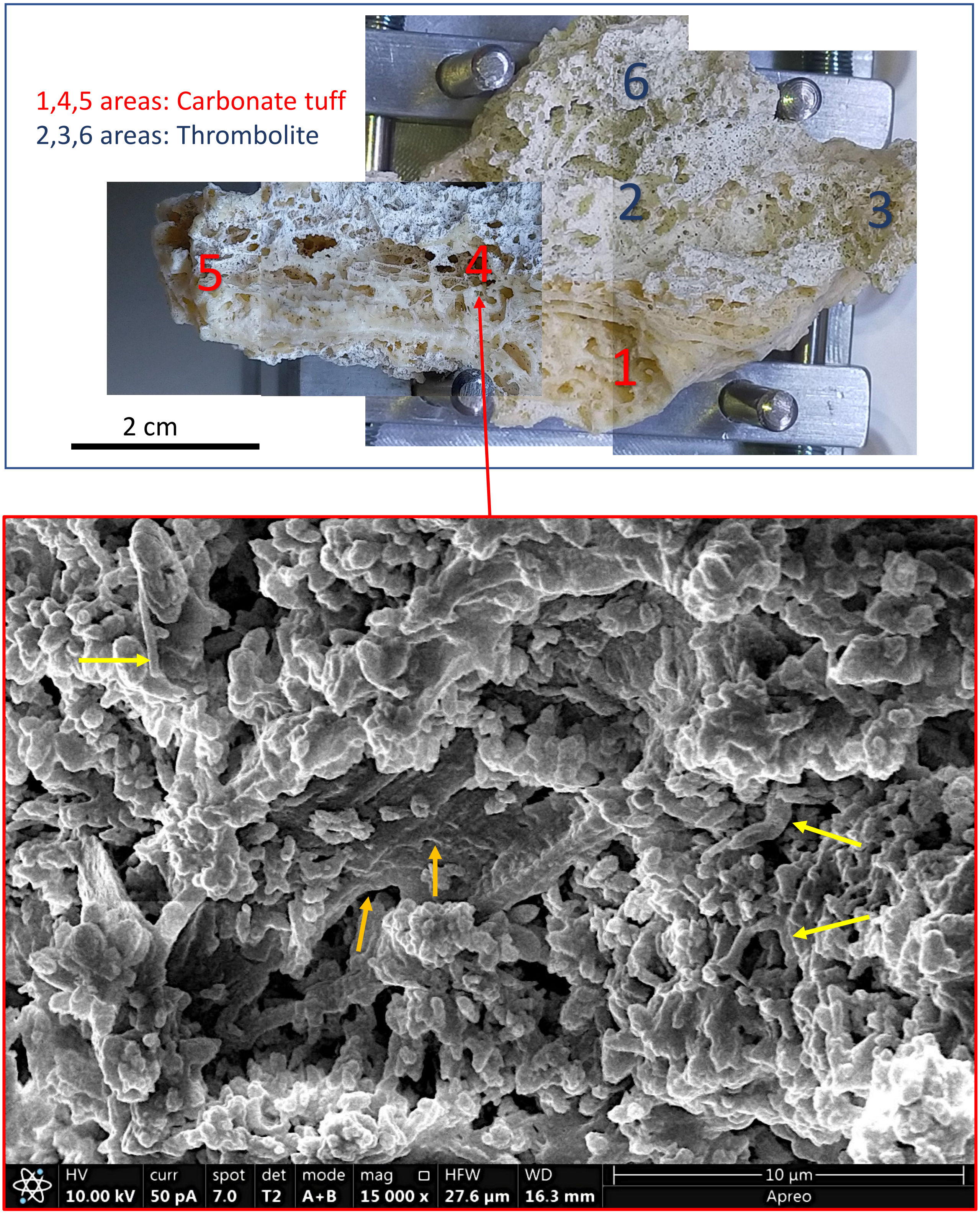

Supplementary Figure 6

Supplementary Table 1. Description of the samples collected in the Salar de Uyuni for this study.

Sample	Coordinates	Location	Sample description	XRD mineral analysis
129	19°24´55.6"S, 68°24´12.8"W	E Pacoma/S Allituma-SW Coipasa (Chile-Bolivia boundary, Coipasa Salar)	Columnar fabric in lacustrine carbonates	$Mg_{0.03}Ca_{0.97}CO_3$
134-1	19°49´06.3"S, 67°50´57.8"W	Chiquini cave (Uyuni Salar)	Speleothem fragments collected from the cave floor showing laminated carbonate with dark patin. They correspond with large pieces of the laminar leafy fabric found in the large speleothem buildings	$Mg_{0.06}Ca_{0.94}CO_3$
134-2				
134-3			Carbonatic tuff with filamentous fabric collected from rocks outcropping in the cave entrance	$Ca_{1.11}Mn_{0.89}(CO_3)_2$ (Kutnahorite), $Mg_{0.1}Ca_{0.9}CO_3$ (Magnesian calcite)
134-4			Complex leafy fabric with internal laminated microstructure and large (> 1 mm thick) filaments	$Mg_{0.129}Ca_{0.871}CO_3$ (Magnesian calcite)

Supplementary Table 2. List of compounds found in the Uyuni samples.

Apolar fraction	Formula	Polar fraction (extracted as TMS derivatives)	Formula	Polar fraction	Formula
Dodecane	C12H26	Decanoic acid	C20H40O2	1-Dodecanol	C12H24O
Tridecane	C13H28	Decanal dimethyl acetal	C12H26O2	1-Tridecanol	C13H26O
Tetradecane	C14H30	Undecanoic acid	C21H42O2	1-Tetradecanol	C14H28O
2,6,10-Trimethyltridecane	C16H34	Undecanal dimethyl acetal	C13H44O2	1-Pentadecanol	C15H30O
Pentadecane	C15H32	Dodecanoic acid	C12H24O2	1-Pentadecanol	C15H30O
Pentadecane, 2-methyl-	C16H34	Dodecanal dimethyl acetal	C14H46O2	2-Pentadecanone, 6,10,14-trimethyl-	C18H36O
Hexadecane	C16H34	Dodecanoic acid, 10-methyl	C13H46O2	1-Pentadecanol	C15H32O
Heptadecane	C17H36	Tridecanoic acid	C13H26O2	1H-Indole-2,3-dione,	C9H7NO3
Pristane	C19H40	Tridecanoic acid, 12-methyl	C14H28O2	1-Hexadecanol	C16H34O
Octadecane	C18H38	Tetradecanoic acid	C14H48O2	1-Heptadecanol	C17H36O
Phytane	C20H42	Tridecanoic acid, 4,8,12-trimethyl-	C16H32O2	Dihydrophytol	C20H42O
Nonadecane	C19H40	Hexadecanal dimethyl acetal	C18H38O2	1-Octadecanol	C18H38O
Benzene, (1-methyldodecyl)-	C17H28	Methyl 13-methyltetradecanoate	C16H32O2	Octadecanone	C18H36O
Eicosene	C20H40	Tetradecanoic acid, 12-methyl-	C15H30O2	1-Nonadecanol	C18H38O
Eicosane	C20H42	Pentadecanoic acid	C15H30O2	Nonadecanone	C19H38O
Heneicosae	C21H44	2-Pentadecanone, 6,10,14-trimethyl-	C18H36O	1-Eicosanol	C20H42O
Docosene	C22H44	Pentadecanoic acid, 14-methyl-	C16H32O2	Eicosanone	C20H40O
Docosane	C22H46	7-Hexadecenoic acid, (Z)-	C16H30O2	1-Heneicosanol	C21H44O
Tricosene	C23H46	9-Hexadecenoic acid, (Z)-	C16H30O2	Heneicosanone	C21H42O
Tricosane	C23H48	Hexadecanoic acid	C16H32O2	Docosanol	C22H46O
Tetracosene	C24H48	10-methyl-hexadecanoate methyl ester	C18H36O2	Docosanone	C22H44O
Tetracosane	C24H50	Hexadecanoic acid, 14-methyl-	C17H34O2	1-Tricosanol	C23H48O
Pentacosane	C25H52	Heptadecanoic acid	C17H34O2	Tricosanone	C23H46O
Hexacosane	C26H54	Octadecane, 1,1-dimethoxy-	C20H42O2	1-Tetracosanol	C24H50O
Heptacosane	C27H56	Hexadecanoic acid, 3,7,11,15-tetramethyl-,	C20H40O2	5-Heptyl-1H-indole-2,3-dione	C10H9NO2
Octacosane	C28H58	9-Octadecenoic acid (Z)-	C18H34O2	Tetracosanone	C24H48O
Squalene	C30H50	10-Octadecenoic acid,	C18H34O2	Pentacosanol	C25H52O
Nonacosane	C29H62	13-Octadecenoic acid,	C18H34O2	Pentacosanone	C25H50O
Triacontane	C30H62	Octadecanoic acid,	C18H36O2	hexacosanol	C26H54O
Hentriacontane	C31H64	Nonadecanoic acid,	C19H38O2	Hexacosanone	C26H52O
Dotriacontane	C32H66	Pentadecanoic acid, 2,6,10,14-tetramethyl-	C19H38O2	Coprostanol	C27H48O
Tritriacontane	C33H68	Methyl 9-eicosenoate	C21H42O2	Heptacosanone	C27H54O
Tetratriacontane	C34H70	Eicosanoic acid	C20H40O2	Octacosanol	C28H58O
Pentatriacontane	C35H72	Heneicosanoic acid	C21H42O2	Cholesterol	C27H46O
Hexatriacontane		Docosanoic acid	C22H44O2	Cholestan-3-ol, (3β,5α)-	C27H48O
		7-Oxodehydroabietic acid	C20H26O3	Cholest-5-en-3-one	C27H44O
		Tricosanoic acid	C23H46O2	1-Triacontanol	C30H62O
		Tetracosanoic acid	C24H48O3	Stigmastanol	C29H52O
		Pentacosanoic acid	C25H50O2	wax ester (lauryl stearate)	C30H60O2
		Cholesta-4,6-dien-3-ol, (3β)-	C27H44O	wax ester (myristyl stearate)	C32H64O2
		Hexacosanoic acid	C26H52O2	3-Hydroxypropyl palmitate,	C19H38O3
		Cholesta-3,5-dien-7-one	C27H42O	Dotriacontanol	C32H66O
		Stigmasta-3,5-dien-7-one	C29H46O	wax ester (cetyl stearate)	C34H68O2
